# Copy Number Variation in Hereditary Non-Polyposis Colorectal Cancer

**DOI:** 10.3390/genes4040536

**Published:** 2013-09-26

**Authors:** Amy L. Masson, Bente A. Talseth-Palmer, Tiffany-Jane Evans, Desma M. Grice, Konsta Duesing, Garry N. Hannan, Rodney J. Scott

**Affiliations:** 1 Information Based Medicine Program, Hunter Medical Research Institute, University of Newcastle, Newcastle, New South Wales, 2305, Australia; E-Mails: c3027685@uon.edu.au (A.L.M.); Bente.Talseth-Palmer@newcastle.edu.au (B.A.T.-P.); Tiffany-Jane.Evans@newcastle.edu.au (T.-J.E.); Desma.Grice@csiro.au (D.M.G.); 2 School of Biomedical Sciences and Pharmacy, Faculty of Health, University of Newcastle, Newcastle, New South Wales, 2308, Australia; 3 CSIRO Preventative Health Flagship and Division of Animal, Food and Health Sciences, North Ryde, New South Wales, 2113, Australia; E-Mails: Konsta.Duesing@csiro.au (K.D.); Garry.Hannan@csiro.au (G.N.H.); 4 Division of Molecular Medicine, Hunter Area Pathology Service, John Hunter Hospital, Newcastle, New South Wales, 2305, Australia

**Keywords:** microsatellite instability (MSI), cancer, DNA repair, diagnostic testing, HNPCC/Lynch Syndrome, copy number variation, affymetrix, array

## Abstract

Hereditary non-polyposis colorectal cancer (HNPCC) is the commonest form of inherited colorectal cancer (CRC) predisposition and by definition describes families which conform to the Amsterdam Criteria or reiterations thereof. In ~50% of patients adhering to the Amsterdam criteria germline variants are identified in one of four DNA Mismatch repair (MMR) genes *MLH1*, *MSH2*, *MSH6* and *PMS2*. Loss of function of any one of these genes results in a failure to repair DNA errors occurring during replication which can be most easily observed as DNA microsatellite instability (MSI)—a hallmark feature of this disease. The remaining 50% of patients without a genetic diagnosis of disease may harbour more cryptic changes within or adjacent to *MLH1*, *MSH2*, *MSH6* or *PMS2* or elsewhere in the genome. We used a high density cytogenetic array to screen for deletions or duplications in a series of patients, all of whom adhered to the Amsterdam/Bethesda criteria, to determine if genomic re-arrangements could account for a proportion of patients that had been shown not to harbour causative mutations as assessed by standard diagnostic techniques. The study has revealed some associations between copy number variants (CNVs) and HNPCC mutation negative cases and further highlights difficulties associated with CNV analysis.

## 1. Introduction

Somewhere between 2% and 5% of all colorectal cancers (CRCs) are classified as hereditary non-polyposis colorectal cancer (HNPCC). Families with germline mutations or complex genomic changes (without structural gene alterations) that render one of four DNA mismatch repair (MMR) genes ineffective compose a subset of HNPCC known as Lynch Syndrome (LS).

The clinical diagnosis of HNPCC is defined by any one of several reiterations of the Amsterdam Criteria, first established in 1990 to enable the identification of the genetic basis of the disease [1]. As such mutations in *MLH1*, *MSH2*, *MSH6* and *PMS2* have been identified to account for all LS families [2,3,4]. Recently, loss of *EPCAM*, has been associated with transcriptional silencing of *MSH2*, and rare epimutations in *MLH1* have also been implicated in LS [5,6].

Despite the definition of HNPCC up to 50% of clinically tested patients with tumours demonstrating microsatellite instability (MSI), the hallmark phenotype of HNPCC, will fail to have any germline mutation identified in any one of the four MMR genes responsible for LS [7,8,9]. This suggests that there are either other genes associated with this disorder or different mechanisms of gene silencing responsible for HNPCC.

Since the sequencing of the human genome it has become apparent that genomic rearrangements are ubiquitous in the population. Genomic duplication or deletion have been shown to encompass large stretches of contiguous DNA and are commonly termed copy number variants (CNVs). As CNVs range from kilobase (Kb) to megabase in size, they may encompass or disrupt functional DNA sequences, result in gene amplification or loss, or alter epigenetic patterning [10]. As such, CNVs have been well documented in their contribution to disease development and variation in disease phenotype [11,12,13,14,15,16].

CNVs have been implicated in the development of many forms of CRC, e.g., germline deletion of two genes, *PTEN* and *BMPR1A* have been identified to be the cause of Juvenile Polyposis (JP) in four unrelated children [17], while genomic deletions in the genes *SMAD4*, *BMPR1A* and *PTEN* result in JP [18] and furthermore, the Leiden Open Variation Database (LOVD) database lists nearly 3,000 mutations in four MMR genes associated with HNPCC, of which many are gains and losses of genomic material [19]. Recent reports specifically examining the association between genomic rearrangements and LS have revealed that loss of a region on chromosome 2 encompassing *EPCAM* appears to be associated with LS [6,20]. The loss of *EPCAM* appears to re-write the epigenetic programming in the region such that the *MSH2* becomes silenced as a result of CpG methylation of the 5' promoter region. This evidence suggests that a proportion of HNPCC families may be accounted for by genomic rearrangements that may not be readily identified using more traditional gene mutation searches.

CNVs are detected using DNA arrays that comprise a series of oligonucleotides that represent evenly distributed markers across the entire genome. As the number of oligonucleotide markers has increased from a few hundred thousand to over five million, CNV resolution has improved such that ever smaller rearrangements can be detected in a single experiment. In this study we have used the Affymetrix Cytogenetic Whole Genome2.7M (Cyto2.7M) array which contains over 400,000 SNP probes and greater than 2.1 million CNV probes (average spacing 1,395 base pairs) to examine the CNV landscape in HNPCC patients and search for CN gains or CN losses which may reside in or in the vicinity of the 22 genes associated with DNA MMR. We also investigated genes and gene expression regulatory elements (microRNAs or miRs) associated with CNVs unique to the HNPCC patients using pathway analysis to determine if they may contribute to disease development.

## 2. Experimental Section

### 2.1. Samples

Genomic DNA samples for the current study were obtained from HNPCC patients who had given informed consent for their DNA to be used for studies into their disease and control DNA samples from the Hunter Community Study (HCS) [21]. DNA was extracted from whole blood by the salt precipitation method [22]. The study was approved by the University of Newcastle’s Human Research Ethics Committee (HREC) and the Hunter New England Human Research Ethics Committee (HNEHREC).

A sample size of 125 HNPCC patients was used for the current study. All HNPCC patients were clinically diagnosed as per the Amsterdam Criteria II [1,23] or the Bethesda Guidelines [24]. All patients had been diagnosed with CRC and were the first individual (proband) of their family to seek genetic testing. The samples were referred for routine clinical diagnostic testing involving screening for mutations in: *MLH1*, *MSH2*, *MSH6* and/or *PMS2*. The mutation screening was performed using Sanger Sequencing and/or Multiplex ligation-dependant probe amplification (MLPA). No mutations were identified in any of the patients used for the current study and are thus considered to be MMR mutation negative. The average age of patients recruited for this study was 52 years of age.

A sample size of 40 controls from the Hunter Community Study (HCS) [21] was used in the current study. Theses samples were from healthy individuals aged >55 years who were cancer free at the time of sample collection.

### 2.2. Genomic Array Analysis

The DNA from the 165 patients and controls was processed on the Affymetrix Cyto2.7M array according to manufacturer’s protocols. CEL files obtained from scanning the Cyto2.7M array were analysed in the proprietary software from Affymetrix, the Chromosome Analysis Suite (ChAS) (Version CytoB-N1.2.0.232; r4280) using NetAffx Build 30.2 (Hg18) annotation. Quality control parameters were optimized and validated using a training set of 20 randomly selected samples (patients and controls). Identified CNV regions within the training set were assessed according to CNV call confidence, probe count, size, wavinessSd and by visual inspection for distinction from normal CN state. In addition, data was visually inspected to identify regions with low density of markers including centromeric and telomeric regions (Supplementary Table 1) which were excluded from analysis across all samples. The resultant thresholds were applied to all samples. Most of the thresholds were more stringent than default settings, aiming to minimize the number of false-positive CNVs being included in the analysis. Briefly, all samples were subject to a series of quality cut-off measures: snpQC >1.1 (assesses quality of SNP probes with respect distances between the distribution of alleles AA, AB and BB alleles and larger differences are associated with an increased ability to identify a genotype; default), mapdQC <0.27 (Median Absolute Pair-wise Difference; assesses quality of CN probes with respect to a reference model file; default) and wavinessSd <0.1 (measure of standard deviation in data waviness; the GC content across the genome correlates with average probe intensities *i.e.*, high GC probes are brighter than low GC probes on average, which creates waves in the data). CNV regions within all samples were then filtered using a set of CNV calling parameters: >90% confidence, autosomes only and a minimum number of 24 probes.

### 2.3. Statistical, Pathway and Annotation Analysis

Refined ChAS CNV counts and CNV size for the patients were compared to the controls using a two tailed un-paired t-test in Graphpad Prism (Version 6) [25]. Gene enrichment analysis was performed using WebGestalt analysis software (Version 2013) [26]. This software was used to assess gene lists derived from the refined CNV results obtained from ChAS according to Gene Ontology (GO) categories, Kyoto Encyclopedia of Genes and Genomes (KEGG) pathways and miR targets. Analysis was performed using hypergeometric statistical method, Benjamini and Hochberg (BH) correction for multiple testing (both default settings) and a biological significance threshold of <0.05 with a minimum of two genes per category required to assess any enrichment. TAM (Tool for Annotations of miRs) (Version 2) [27] software was used to annotate miRs according to miR family, cluster, function, Human miR associated disease categories (HMDD) and tissue specificity. Annotations were performed using the following parameters: all miRs in the TAM database were used as a background; to identify meaningful categories we looked at miR over-representation in all categories and analysis was limited to at least one miR in a given category. Enrichment analysis for miRs categories was conducted using hypergeometric testing and *p* values were corrected according to Bonferroni correction for multiple testing.

## 3. Results and Discussion

Recent studies have reported CNV’s as relevant contributors to human diversity and cancer susceptibility [28,29,30]. This study further defines the contribution of CNVs to disease risk in HNPCC.

### 3.1. Resolution

**Figure 1:**
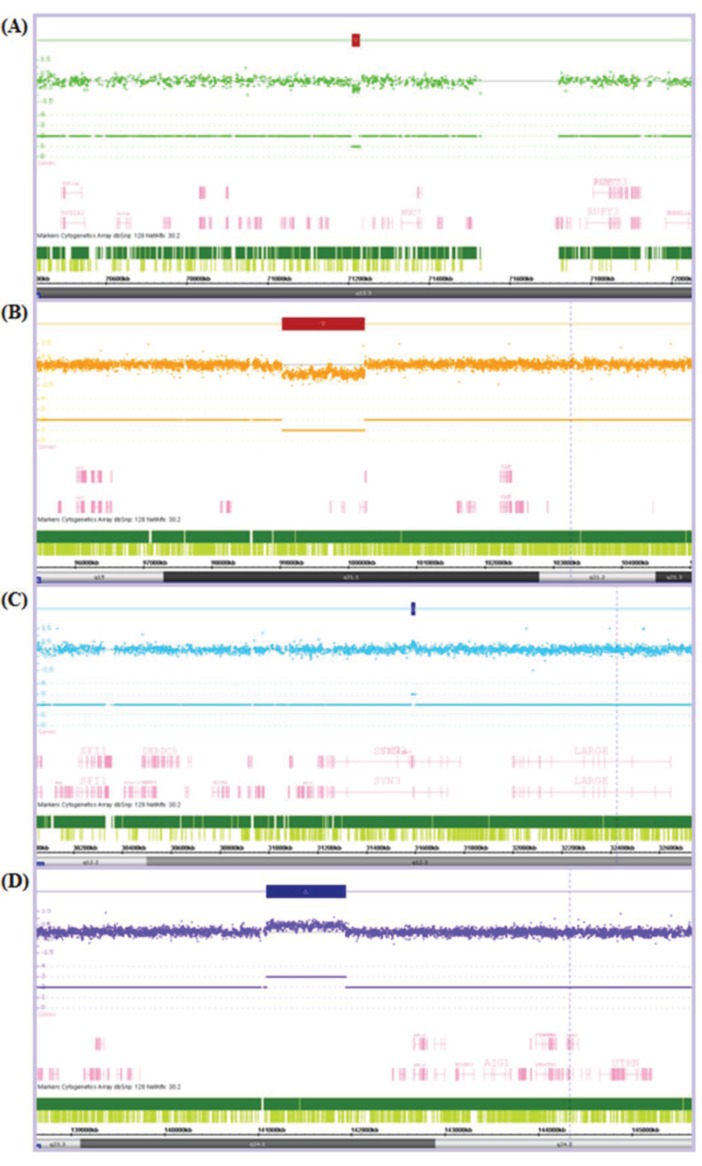
Chromosome Analysis Suite (ChAS) output showing examples of (**A**) a small copy number (CN) loss of 17.8 Kb; (**B**) a large CN loss of 1,205.7 Kb; (**C**) a small CN gain of 14.8 Kb; and (**D**) a large CN gain of 843.3 Kb. The Log2Ratio represents the relative fluorescence of each probe (dot) across the genome (from left to right). The fluorescence is reduced in regions of CN loss and increased in regions of CN gain. This is indicated by a CN loss (red box) or CN gain (blue box) over the affected region and the resultant CN state noted below e.g., there is only one of the two alleles present in each CN loss and an extra allele present in each of the CN gains.

Refinement of ChAS thresholds resulted in the final analysis of CNVs ranging from a minimum of 8.4 Kb to a maximum of 2,722.5 Kb in size (see Figure 1 for examples). CN gains ranged from 8.4 Kb to 2,722 Kb in patients and 14.8 Kb to 1,076.2 Kb in controls while CN losses ranged from 17.8 Kb to 529.2 Kb in patients and 16.8 Kb to 1,205.7 Kb in controls. As such we cannot rule out the potential involvement of CNVs below the level of detection of the Cyto2.7M array, in the aetiology of HNPCC.

### 3.2. CNV Detection

Analysis of Cyto2.7M array data identified a total of 543 CNVs in the 165 patients and controls utilized in this study (Table 1). Total counts of CNVs observed in the 125 HNPCC patients corresponded to 439 CNV events compared to 104 events in the 40 controls. The mean number of CNVs identified per sample did not significantly differ between patients and controls (3.51 CNVs per patient and 2.60 CNVs per control, *p* = 0.2980). Consistent with a recent report looking at CNVs in hereditary breast cancer, similar counts of CNVs detected between patients and controls have been suggested to reflect a lack of genomic instability in the genomes of patients screened [31,32]. The mean CNV affected genome per sample did not differ between patients and controls either (284.07 Kb patients and 295.52 Kb controls, *p = 0.9121*). However, the mean size of a CNV differed significantly between patients and controls (70.08 Kb in patients and 106.57 Kb in controls, *p* = 0.0165). The exact reason why we observe this difference is unclear however it may be a function of the number of samples in each group.

**Table 1:** Summary of copy number variants (CNV) results obtained from the Cyto2.7M array analysed in ChAS.

		CNV Count			CNV Size (Kb)		
Participants		Total CNVs per group	Median CNVs per sample	Mean CNVs per sample	Total CNV affected genome per group	Mean total CNV affected genome per sample	Mean size of a CNV
**Patients**	125	439	2	3.51	35,508.53	284.07	70.08
**Controls**	40	104	2	2.60	11,820.75	295.52	106.57
***p***	−	−	−	0.2980	−	0.9121	0.0165 *

* statistically significant.

### 3.3. MMR Gene Interrogation

CNVs in patients and controls were interrogated for CN gains and losses residing in or in the vicinity of (50 Kb upstream to 50 Kb downstream) the 22 genes (*EXO1*, *LIG1*, *MLH1*, *MLH3*, *MSH2*, *MSH3*, *MSH6*, *PCNA*, *PMS1*, *PMS2*, *POLD1*, *POLD2*, *POLD3*, *POLD4*, *RFC1*, *RFC2*, *RFC3*, *RFC4*, *RFC5*, *RPA1*, *RPA2* and *RPA3*) in the MMR pathway (see Table 2). We aimed to identify CNVs which could potentially contribute to disease development directly (e.g., disruption of functional gene sequences or promoter region inactivation) and via other mechanisms, including the alteration to epigenetic marks (as seen with the transcriptional silencing of *MSH2* through a CN loss in *EPCAM* in several LS patients, described previously [6,20]).

No CN gains or losses were identified within the defined search region for any of the 22 genes in the MMR pathway for all samples utilized in this study, patients and controls. We cannot however rule of the possibility for CNVs residing in these regions which are smaller than the resolution of detection provided by this array (<8.4 Kb).

**Table 2:** Regions searched for CN gains and CN losses in and in the vicinity of (±50 Kb) of the 22 genes in the mismatch repair (MMR) pathway. Chromosomal position of gene (start and end), gene size and search region (search start and search end) is noted.

Gene	Chr.	Start (bp)	End (bp)	Size (Kb)	Search start (bp)	Search end (bp)
***EXO1***	1	240,078,157	240,119,671	42	240,028,157	240,169,671
***RPA2***	1	28,090,635	28,113,823	23	28,040,635	28,163,823
***MSH2***	2	47,783,766	47,563,864	80	47,733,766	47,613,864
***MSH6***	2	47,863,724	47,887,596	24	47,813,724	47,937,596
***PMS1***	2	190,357,055	190,450,600	94	190,307,055	190,500,600
***MLH1***	3	37,009,982	37,067,341	57	36,959,982	37,117,341
***RFC4***	3	187,990,375	188,007,178	17	187,940,375	188,057,178
***RFC1***	4	38,965,470	39,044,390	79	38,915,470	39,094,390
***MSH3***	5	79,986,049	80,208,390	222	79,936,049	80,258,390
***PMS2***	7	5,979,395	6,015,263	36	5,929,395	6,065,263
***POLD2***	7	44,120,810	44,129,672	9	44,070,810	44,179,672
***RFC2***	7	73,283,767	73,306,674	23	73,233,767	73,356,674
***RPA3***	7	7,643,099	7,724,763	82	7,593,099	7,774,763
***POLD3***	11	73,981,276	74,031,413	50	73,931,276	74,081,413
***POLD4***	11	66,875,594	66,877,593	2	66,825,594	66,927,593
***RFC5***	12	116,938,890	116,954,422	16	116,888,890	117,004,422
***RFC3***	13	33,290,205	33,438,695	148	33,240,205	33,488,695
***MLH3***	14	74,550,219	74,587,988	38	74,500,219	74,637,988
***RPA1***	17	1,680,022	1,749,598	70	1,630,022	1,799,598
***LIG1***	19	53,310,514	53,365,372	55	53,260,514	53,415,372
***POLD1***	19	55,579,404	55,613,083	34	55,529,404	55,663,083
***PCNA***	20	5,043,598	5,055,268	12	4,993,598	5,105,268

### 3.4. Occurrence and Distribution of CNVs in Patients and Controls

Of the total 104 CNVs identified in controls, 34 CNVs contained genomic regions that were common to genomic regions identified in patients (Supplementary Table 2). A total of 70 CNVs were unique to the controls of which 47 (67.14%) were associated with genes (Supplementary Table 3).

Of 439 CNVs identified in patients, 53 CNVs contained genomic regions that were common to genomic regions identified in controls (Supplementary Table 4). Three hundred and eighty six CNVs were unique to the patients population of which 207 (53.63%) were associated with genes (Supplementary Table 5).

From the 207 unique CNVs associated with genes identified in the patients, 9 were identified in patients that did not overlap any CNVs in controls but affected the same gene even in multiple patients (*ARPP-21*, *C7orf10*, *KIAA1217*, *LINGO2*, *MACROD2* and *NKAIN2*). A total of 60 genes associated with 131 CNVs were identified in multiple individuals (as shown in Table 3). Fifty two genes were affected by a CNV in two individuals; five genes were affected by a CNV in three individuals (*IGSF11*, *GK5*, *XRN1*, *NAMPT* and *LCP1*); and three genes were affected by a CNV in four individuals (*CTNNA3*, *NRG3* and *LOC642597*).

**Table 3:** Genes associated with unique CNVs (compared to controls) identified across multiple patients. Number of CNV events in which gene (s) have been identified and if they were a CN gain or loss.

Type	2 CNV events				3 CNV events	4 CNV events
**Gains**	*ADARB2*	*DEFB125*	*ITGA1*	*PSG8*	*GK5*	*LOC642597*
	*APC*	*DEFB126*	*KIAA1680*	*RBCK1*	*IGSF11*	
	*ARHGAP19*	*DEFB127*	*LATS2*	*RNF125*	*LCP1*	
	*B2M*	*DEFB128*	*MLL*	*RNF138*	*XRN1*	
	*BBOX1*	*DEFB129*	*MSI2*	*SOX12*	*NAMPT*	
	*C10orf139*	*DEFB132*	*NRSN2*	*TBC1D20*		
	*C14orf23*	*EPHA7*	*NXPH1*	*TFG*		
	*C20orf96*	*FAM134B*	*ODZ4*	*TRIB3*		
	*C3orf33*	*FOXG1*	*PELO*	*TRIM69*		
	*CNTN5*	*GPR128*	*PHC3*	*WDR37*		
	*CNTNAP2*	*GPR160*	*PRKCI*	*ZCCHC3*		
	*CSNK2A1*	*GYPE*	*PSG10*	*ZMYND11*		
**Losses**	*CNTN4*					*CTNNA3*
	*DCDC1*					
	*PPP2R3C*					
**Both**						*NRG3*

While this study has not investigated further the contribution of any one of these CNVs to disease development, previous studies have reported the involvement of several of the genes influenced by one or more CNVs in CRC: L-plastin (subunit *LCP1*) has been shown to be unregulated in various solid human tumours and is also known to contribute to CRC progression via its involvement in cell proliferation and invasion and consequently metastasis [33,34,35]; alpha-catenin (subunit *CTNNA3*) has been reported to show reduced expression in CRC cell lines which has been suggested to facilitate metastasis [36], while another study has reported increased expression of alpha-catenin during adenoma formation via the negative regulation of beta-catenin signalling [37]; the tumour suppressor gene *APC* has been unequivocally associated with the CRC and Familial adenomatous polypsis (FAP) [38,39,40]; and furthermore, expression of *IGSF11* has been reported to be elevated in CRC cells lines and may represent a target for cancer immunotherapy [41]. Future studies are required to validate and investigate the role of the CNVs identified in our study for their potential contribution in the development of HNPCC.

Of the 386 CNVs identified unique to the patients, of these regions 56.5% of them have been previously reported in the Database of Genomic Variance (DGV). Fifty nine CNVs contained genomic regions which were identified in multiple patients (Table 4). A total of 15 genomic regions were identified in two patients; five common genomic regions were identified in three patients, located on chromosomes 3, 5, 9, 11 and 12; and one genomic region was identified in four patients on chromosome 16. Two other CNVs were also shown to be common to five patients on chromosomes 3 and 5. Additional studies are required to investigate the sequence content of these regions to identify if novel contributors to disease development may reside in these regions.

**Table 4:** Genomic regions associated with unique CNVs (compared to controls) identified across multiple among patients. Note CNV frequency and CNV type; CNV location (chromosome, start bp and end bp) and size; as well as the confidence score associated with CNV call and the number of probes used to call the CNV are also noted.

Chr.	Start (bp)	End (bp)	Size (Kb)	Conf	Probes
**2 CNV gains**					
3	189,058,439	189,098,718	40.28	0.93	31
3	189,069,317	189,088,009	18.69	0.94	26
4	44,664,798	44,699,744	34.95	0.92	41
4	44,664,798	44,699,744	34.95	0.90	41
8	120,414,388	120,438,172	23.78	0.91	27
8	120,419,721	120,451,773	32.05	0.91	30
11	29,547,229	29,593,722	46.49	0.90	39
11	29,547,756	29,593,722	45.97	0.91	37
16	25,330,672	25,438,375	107.70	0.92	46
16	25,330,672	25,438,375	107.70	0.92	46
**3 CNV gains**					
3	19,014,033	19,041,376	27.34	0.90	31
3	19,016,875	19,041,376	24.50	0.91	28
3	19,016,875	19,041,376	24.50	0.91	28
5	59,744,695	59,807,906	63.21	0.92	52
5	59,744,695	59,811,770	67.08	0.93	54
5	59,749,693	59,807,906	58.21	0.92	51
9	103,982,826	104,016,588	33.76	0.91	27
9	103,982,826	104,017,715	34.89	0.90	28
9	103,991,205	104,017,715	26.51	0.91	26
11	15,765,333	15,791,331	26.00	0.90	30
11	15,770,233	15,796,302	26.07	0.92	30
11	15,776,946	15,795,665	18.72	0.91	24
12	16,469,855	16,503,960	34.11	0.91	33
12	16,469,855	16,503,960	34.11	0.91	33
12	16,476,470	16,506,851	30.38	0.92	33
**4 CNV gains**					
16	63,364,955	63,389,659	24.70	0.91	33
16	63,369,029	63,389,029	20.00	0.92	30
16	63,369,960	63,388,189	18.23	0.90	28
16	63,371,038	63,397,352	26.31	0.92	38
**5 CNV gains**					
5	116,651,923	116,698,621	46.70	0.91	36
5	116,655,439	116,692,153	36.71	0.92	27
5	116,656,039	116,695,730	39.69	0.91	29
5	116,660,694	116,697,347	36.65	0.93	28
5	116,660,694	116,693,035	32.34	0.91	24
**2 CNV losses**					
1	82,801,000	82,821,932	20.93	0.93	31
1	82,801,000	82,821,932	20.93	0.94	31
2	22,087,558	22,261,901	174.34	0.91	110
2	22,087,558	22,261,901	174.34	0.93	110
2	215,167,158	215,204,595	37.44	0.93	49
2	215,167,158	215,204,595	37.44	0.91	49
3	6,562,398	6,603,706	41.31	0.94	42
3	6,562,398	6,603,706	41.31	0.93	42
3	166,523,809	166,565,186	41.38	0.95	39
3	166,525,250	166,565,186	39.94	0.93	38
5	61,460,851	61,504,678	43.83	0.95	31
5	61,460,851	61,504,678	43.83	0.93	31
7	92,319,307	92,343,906	24.60	0.94	26
7	92,319,307	92,343,906	24.60	0.94	26
9	104,331,902	104,396,632	64.73	0.96	35
9	104,331,902	104,396,632	64.73	0.96	35
**5 CNV losses**					
3	177,370,126	177,396,832	26.71	0.93	26
3	177,370,126	177,396,832	26.71	0.94	26
3	177,370,126	177,396,832	26.71	0.96	26
3	177,370,126	177,399,625	29.50	0.93	27
3	177,370,126	177,396,832	26.71	0.93	26
**2 CNV gain and loss**					
7 *	110,748,452	111,047,157	298.71	0.93	291
7 **	111,007,466	111,052,498	45.03	0.94	25
3 **	21,228,980	21,313,310	84.33	0.90	88
3 *	21,273,619	21,339,035	65.42	0.92	62

* loss ** gain.

### 3.5. Pathway Analysis

WebGestalt [26] pathway analysis software was then used to compare a list of 317 genes associated with CNVs uniquely identified across all patients (compared to controls) to all genes in the human genome (Supplementary Table 6). Enrichment analysis of KEGG pathways and miR targets was conducted. In relation to the control population we also undertook a similar analysis which revealed three pathways, a tight junction pathway and two related pathways, an oocyte meiosis and a progesterone mediated oocyte maturation related pathway. Of particular note was the absence of any cancer related pathway in the control population suggests that those identified in the patient population are likely to be involved in some aspect of malignancy.

KEGG analysis revealed a total of 18 significant pathways in which genes uniquely identified in the patients were enriched (Table 5). The most significant pathways identified included those of the carbohydrate digestion and absorption (*p* = 0.0012); starch and sucrose metabolism (*p* = 0.0017); and metabolic pathways (*p* = 0.0023) affecting a total of 11 patients. Previous studies have suggested that changes occurring in metabolic pathways are commonly observed during carcinogenesis and tumour growth [42,43]. In the context of this study, these results suggest the potential existence of a germline predisposition in the affected patients which lead to metabolic conditions that promote disease development. The tight junction pathway (*p* = 0.0058) and neurotrophin signalling pathway (*p* = 0.0058) were also identified to be enriched and have been shown to play a role in gut permeability and motility [44,45,46]. These pathways have been well documented for their contribution to CRC [47,48,49,50]. It is interesting to also note among the enriched KEGG pathways the prostate cancer pathway (*p* = 0.0251) and endometrial cancer pathway (*p* = 0.0251) also featured and represent two cancers commonly arising in the general population and in the setting of HNPCC/LS [51,52,53]. Overall, our KEGG results suggest the existence of genetic risk factors which may act to promote the development of cancer.

**Table 5:** Enriched Kyoto Encyclopedia of Genes and Genomes (KEGG) pathways from genes identified from CNVs unique to patients.

KEGG Pathway	Genes in Pathway	Observed	Expected	*p*
Carbohydrate digestion and absorption	44	5	0.32	0.0012
Starch and sucrose metabolism	54	5	0.4	0.0017
Metabolic pathways	1,130	21	8.31	0.0023
Salivary secretion	89	5	0.65	0.0058
Tight junction	132	6	0.97	0.0058
Neurotrophin signaling pathway	127	6	0.93	0.0058
Propanoate metabolism	32	3	0.24	0.017
Valine, leucine and isoleucine degradation	44	3	0.32	0.0251
Prostate cancer	89	4	0.65	0.0251
Ribosome biogenesis in eukaryotes	80	4	0.59	0.0251
ErbB signaling pathway	87	4	0.64	0.0251
mRNA surveillance pathway	83	4	0.61	0.0251
Terpenoid backbone biosynthesis	15	2	0.11	0.0285
Malaria	51	3	0.37	0.0293
Endometrial cancer	52	3	0.38	0.0293
Glycerolipid metabolism	50	3	0.37	0.0293
Olfactory transduction	388	8	2.85	0.0346
beta-Alanine metabolism	22	2	0.16	0.0439

Enrichment analysis for targets of miRs identified 65 significant regions within the 3' UTR of the CNV impacted genes unique in the patients. We identified *in silico* 114 miRs (Supplementary Table 7) that target these genes regions with over 35% of these having previously been reported to have associations with CRC [54,55,56,57,58,59,60,61,62,63,64,65,66,67,68,69,70,71,72,73,74,75,76]. Of the top 10 most significant regions, 40% of the miRs we identified using our approach have been associated with CRC (mi*R-141*, mi*R-15A*, mi*R-15B*, mi*R-18A*, mi*R-200A*, mi*R-200B*, mi*R-203*, mi*R-32*, mi*R-429* and mi*R-92*). Overall, our miR enrichment analysis supports reported findings on miR involvement in CRC. Confirmation of the respective miRs by functional analysis is required to unequivocally demonstrate their role in HNPCC.

In summary the results obtained from the pathway analysis suggest that many of the genes associated with CNVs uniquely identified in patients are associated with carcinogenesis, tumour growth and disease susceptibility and may be factors in the development of CRC.

### 3.6. MicroRNA Annotation

From the 317 genes affected by a CNV in the patient cohort we identified, using pathway analysis, 65 genes that enriched 3' UTR microRNA target regions. These 65 binding regions were associated with 114 proposed regulatory microRNAs. TAM (a Tool for Annotations of miRs) software [27] was then used to identify meaningful miR categories among the 114 miRs that target significantly enriched 3' UTR regions identified in the patients from previous pathway analysis (Table 6). We identified a total of 261 miR categories: 22 families, 33 clusters, 39 functional categories, 162 HMDD and 5 tissue specificity categories. It was identified that miRs were enriched in the family category miR-17 (*p* = 0.0011). A total of 10 functional categories were enriched including those associated with onco-miRs (*p* = 0.0264), processes of apoptosis (*p* = 0.0291) and cell-cycle (*p* = 0.0406). For the HMDD category miRs were enriched in various forms of cancer, with cancer enrichment alone accounting for 80% of the most significant findings.

**Table 6:** Summary of significant findings from miR annotation analysis in TAM (A Tool for Annotations of miRs) software.

Category	# sub-categories	# of Sig. Categories	Significant Findings
Family	22	1	miR-17
Cluster	33	0	−
Function	39	8	Onco-miRs, Apoptosis, Cell cycle related
HMDD	162	20	Cancer (80%), Cardiovascular (4%), Infection (4%) and Psychological disorders (10%)
Tissue	5	1	Placenta

In the context of our study it was reassuring to note the presence of adenocarcinoma (*p =* 0.0000155) and colorectal neoplasm’s (*p* = 0.0253) among the cancers enriched in miR annotation. Cardiovascular diseases (4%), psychological disorders (10%) and infection (4%) represented the minority of other significant finds (all with *p* < 0.0047). According to tissue specificity, the placenta represented the most significant miR enriched tissue (*p* = 0.00001284). Previous studies have suggested that processes of angiogenesis and vascularisation occurring during placental development in pregnancy are also present during tumour development and this has been observed in CRC [77,78,79].

Overall the results obtained from the TAM analysis suggest that the 114 miRs associated with the 3' UTR regions significantly identified in the patients may stimulate processes leading to carcinogenesis which is consistent with what we expect to find in these cancer patients [80].

### 3.7. CNV Burden

A study by Girirajan and Eichler [81] has suggested that the severity of disease may be explained by the overall burden CNVs place on an individual’s genome where increased sensitivity to developing disease is correlated with increased CNV burden and furthermore that variation in CNV burden will result in phenotype variation in patients. In a recent study looking at both HNPCC MMR mutation negative and MMR mutation positive patients, we observed an increased average size of CNVs in patients tested and suggested that this was related to an increased genomic burden [82]. An increased CNV burden was not observed among the patients utilized in the current study, though we did detect a decreased mean size of CNVs in patients compared to controls. Importantly, the current study compared 125 patients and 40 controls whereas the recent HNPCC study compared 96 patients and 384 controls [82]. We suggest that discrepancies in these findings are likely to be related to the inequity of sample populations between studies, the limited number of controls used in the current study, the type of array used (noting differences in both the array coverage and density), as well as the algorithm used by analysis software may all contribute to variation in the observed results [83,84,85].

### 3.8. CNV Bias

CNV analysis has suffered from a lack of standardization in analytical techniques used for data mining. The Hidden Markov Model (HMM) and Circular Binary Segmentation (CBS) represent the algorithms utilized to develop CNV calling programs that have been reported to be the most efficient [83,84,85]. Furthermore, using algorithms developed for a specific data type has been shown to perform better in CNV calling compared to platform-independent software algorithms [86]. The robustness of software algorithms, batch effects, and population stratification will therefore influence the accuracy of calls made to segmented data and hence the reliability of CNV calls and CNV boundary descriptors derived from arrays [83,84,85]. The Cyto2.7M array was chosen for use in the current study as at the time it provided the greatest density and most even genomic coverage of any CNV arrays. All data was analysed through ChAS which uses a HMM-based algorithm and was specifically developed to use with the Cyto2.7M array data.

To improve our predictive accuracy we did consider merging other CNV data sets that were at our disposal [82], but this would result in a significant loss of information since the our original data set was created using a SNP based array which did not have even coverage across the genome whereas the one used in this report was an oligo-based array with uniform coverage. Any merged dataset would lose a significant amount of information.

Finally, a recent report suggesting the use of a minimum of three different algorithms when conducting CNV association analysis, this was not possible due to limitations in the data generated by the array used in this study [87].

## 4. Conclusions

We were unable to identify any DNA mismatch repair genes targeted by CNVs that may contribute to a significant proportion of HNPCC patients recruited into this study. We did identify several genomic regions that were altered in multiple unrelated HNPCC patients that could potentially be associated with disease risk. The genomic regions encompassed by these CNVs warrant further study to define precisely their role in disease development. We could not rule out the existence of CNVs, smaller than the limits of detection provided by this array, from involvement in the aetiology of HNPCC.

Pathway analysis was thus utilized to identify possible common pathways associated with the heterogeneous outcomes of the analysis. We identified a total of 317 genes impacted by CNVs uniquely identified in patients (compared to controls). Results from KEGG pathway analysis identified the enrichment of pathways involved in metabolism, and these are known to be required for cancer development. It is likely that these loci may contribute to CRC disease risk in the affected individuals. miR enrichment analysis has further highlighted a series of miRs which are suggested to contribute to carcinogenesis. It was found that over 40% of these miRs had been previously reported to play a role in CRC development. As such we have shown that CNV altered genes are over represented in pathways leading to carcinogenesis, tumour growth and disease susceptibility, including CRC. The genes driving pathway enrichments require further investigation to elucidate their precise role in disease development.

The annotation of 114 miRs (reported in the pathway analysis) identified significant functional miR categories associated with cancer, including specifically adenocarcinomas and colorectal neoplasms. Placental tissue was identified to be among the tissues most significantly enriched with the miR looked at in this investigation. We speculate that processes of angiogenesis and vascularisation necessary for placental development are also present during tumour formation including those observed in CRC. As such the genes associated with CNVs we have identified are targeted by miRs which are implicated in various processes leading to malignancy. We conclude that while we have not shown direct consequences of miRs interacting with our CNV altered genes, the separate effects of aberrant miR expression and CNVs impacting on genes that such miRs target may have similar consequences.

Overall the results of this study provide some evidence of CNV involvement in the aetiology of HNPCC and furthermore reinforce that CNV probe arrays compared to SNP arrays appear to be of limited utility for CNV detection.

## Supplementary Files

Supplementary File 1 Supplementary Information (PDF, 908 KB)
