# Supplementary material for: Copy Number Variation in Hereditary Non-Polyposis Colorectal Cancer
Source: Genes (Basel). 2013 Sep 26;4(4):536–55. doi: 10.3390/genes4040536 (PMC3927572; doi:10.3390/genes4040536)
Supplement: Supplementary File 1 — Supplementary Information (PDF, 908 KB) [file genes-04-00536-s001.pdf]

Supplementary Information

Table S1. Regions of CNV data excluded from CNV analysis due to poor density of probe coverage.

Region	Centromere		Telomere	
**Chr.**	p	q	p	q
**1**	p11.1, p11.2, p12, p13.1	q11, q12, q21.1	p36.33, p36.32, p36.31, p36.23, p36.22, p36.21	q44
**2**	p11.1, p11.2	q11.1	p25.3	q37.3
**3**	p11.1	q11.1, q11.2	none excluded	q29
**4**	p11	q11	p16.1, p16.2, p16.3	q35.2, q35.1
**5**	p12	q11.1	p15.33	q35.3, q35.2, q35.1
**6**	p11.1	q11.1	p25.3, p35.2	q27
**7**	p11.1	q11.1, q11.21	p22.3	q36.3
**8**	p11.1	q11.1	p23.3, p23.2, p23.1	q24.3
**9**	p11.1, p11.2	q11, q12, q13	p24.3	q34.3, q34.2, q34.13, q34.12, q34.11
**10**	p11.1	q11.1, q11.21, q11.22	none excluded	q26.3
**11**	p11.11, p11.12	q11	p15.5	q25
**12**	p11.1, p11.21	q11	p13.33, p13.32	q24.33, q24.32, q24.31
**13**	no probes	q11	no probes	q34
**14**	no probes	q11.1, q11.2	no probes	q32.33
**15**	no probes	q11.1, q11.2, q12, q13.1, q13.2, q13.3	no probes	q26.3
**16**	p11.1, p11.2	q11.1, q11.2	p13.3	q24.3, q24.2, q24.1
**17**	none excluded	none excluded	p13.3	q25.3
**18**	p11.1, p11.21	q11.1	none excluded	q23
**19**	p11	q11	p13.3	q13.43
**20**	p11.1, p11.21	q11.1, q11.21	none excluded	q13.33
**21**	no probes	q11.1, q11.2	no probes	q22.3
**22**	no probes	q11.1, q11.21, q11.22	no probes	q13.33, q13.32

Table S2. List of 34 CNVs identified in controls that are in-common with CNVs identified in patients. Chromosomal position (start and end), CNV size (Kb), CN type, gene(s) affected and number of probes detecting the region are noted for all CNVs.

Chr.	Start (bp)	End (bp)	Size (Kb)	Type	Conf	Genes	Probes
**2**	57,252,457	57,293,667	41.21	Gain	0.97		30
**2**	57,252,457	57,293,667	41.21	Gain	0.97		30
**3**	174,722,068	174,773,568	51.5	Gain	0.91	*NLGN1*	51
**3**	174,722,068	174,772,293	50.225	Gain	0.92	*NLGN1*	50
**3**	174,722,068	174,772,293	50.225	Gain	0.92	*NLGN1*	50
**3**	174,722,313	174,772,293	49.98	Gain	0.92	*NLGN1*	48
**3**	174,722,313	174,772,293	49.98	Gain	0.90	*NLGN1*	48
**4**	66,267,516	66,304,981	37.465	Gain	0.91		37
**4**	66,271,495	66,311,088	39.593	Gain	0.91		40
**4**	162,167,234	162,223,002	55.768	Gain	0.90		45
**4**	162,167,234	162,223,002	55.768	Gain	0.90		45
**4**	162,172,629	162,223,002	50.373	Gain	0.93		44

Table S2. *Cont.*

Chr.	Start (bp)	End (bp)	Size (Kb)	Type	Conf	Genes	Probes
**5**	32,144,171	32,202,727	58.556	Gain	0.95	*PDZD2, GOLPH3*	55
**5**	32,145,347	32,202,727	57.38	Gain	0.93	*PDZD2, GOLPH3*	53
**6**	124,475,557	124,512,504	36.947	Gain	0.92	*NKAIN2*	51
**6**	124,476,194	124,512,504	36.31	Gain	0.94	*NKAIN2*	50
**6**	124,476,733	124,512,504	35.771	Gain	0.93	*NKAIN2*	49
**8**	57,211,297	57,260,396	49.099	Gain	0.94	*PLAG1*	61
**8**	57,217,324	57,261,033	43.709	Gain	0.91	*PLAG1*	58
**12**	7,895,002	8,009,418	114.416	Gain	0.91	*SLC2A14, SLC2A3*	30
**12**	7,895,002	7,985,800	90.798	Gain	0.90	*SLC2A14, SLC2A3*	28
**12**	7,895,002	8,009,418	114.416	Gain	0.90	*SLC2A14, SLC2A3*	30
**4**	21,656,799	21,681,104	24.305	Loss	0.90		29
**4**	21,656,799	21,681,104	24.305	Loss	0.90		29
**4**	35,051,978	35,088,582	36.604	Loss	0.95		33
**5**	99,035,945	100,241,677	1205.732	Loss	0.94	*LOC100133050, FAM174A, ST8SIA4*	785
**5**	104,461,414	104,529,373	67.959	Loss	1.00	*RAB9P1*	40
**5**	104,461,414	104,529,373	67.959	Loss	0.94	*RAB9P1*	40
**7**	8,794,054	8,831,295	37.241	Loss	0.96		35
**7**	141,967,332	142,005,798	38.466	Loss	0.91		40
**8**	137,751,665	137,919,630	167.965	Loss	0.92		56
**8**	137,751,665	137,919,630	167.965	Loss	0.95		56
**15**	85,626,838	85,671,184	44.346	Loss	0.95		48
**20**	14,729,522	14,774,064	44.542	Loss	0.95	*MACROD2*	57

Table S3. List of 70 CNVs identified in controls that are unique compared to the CNVs identified in patients. Chromosomal position (start and end), CNV size (Kb), CN type, gene(s) affected and number of probes detecting the region are noted for all CNVs.

Chr.	Start (bp)	End (bp)	Size (Kb)	Type	Conf	Genes	Probes
**1**	49,726,057	49,870,951	144.894	Loss	0.93	*AGBL4*	149
**3**	35,778,207	35,916,128	137.921	Gain	0.93	*ARPP-21*	131
**10**	46,137,934	47,214,122	1076.188	Gain	0.95	*BMS1P5, FAM35B, SYT15, GPRIN2, PPYR1, LOC728643, ANXA8, ANXA8L1, FAM25G, FAM25B, FAM25C, LOC642826, FAM35B2, ANTXRL*	141
**22**	30,473,467	30,506,249	32.782	Gain	0.95	*C22orf30, DEPDC5*	29
**3**	54,994,537	55,017,549	23.012	Gain	0.91	*CACNA2D3*	32
**7**	39,966,237	40,136,268	170.031	Gain	0.90	*CDC2L5*	151
**7**	40,063,698	40,167,873	104.175	Gain	0.93	*CDC2L5, C7orf11, C7orf10*	57
**6**	21,250,491	21,273,107	22.616	Gain	0.90	*CDKAL1*	40
**2**	124,812,530	124,830,318	17.788	Loss	0.96	*CNTNAP5*	24
**8**	139,733,471	139,749,887	16.416	Gain	0.91	*COL22A1*	26
**15**	81,040,928	81,262,556	221.628	Gain	0.90	*CPEB1, AP3B2, SCARNA15, FSD2*	141
**8**	19,412,976	19,476,888	63.912	Loss	0.92	*CSGALNACT1*	81
**7**	133,163,943	133,185,357	21.414	Gain	0.91	*EXOC4*	26
**3**	75,510,347	75,718,206	207.859	Loss	0.91	*FAM86D*	49

Table S3. *Cont.*

Chr.	Start (bp)	End (bp)	Size (Kb)	Type	Conf	Genes	Probes
**7**	151,383,678	151,527,919	144.241	Gain	0.91	*GALNT11*, *MLL3*	123
**6**	142,630,512	142,683,762	53.25	Gain	0.93	*GPR126*	52
**2**	74,798,769	75,093,133	294.364	Gain	0.90	*HK2*, *POLE4*	213
**6**	15,456,927	15,474,911	17.984	Loss	0.91	*JARID2*	26
**8**	73,761,439	73,801,718	40.279	Gain	0.91	*KCNB2*	49
**10**	24,567,203	24,585,138	17.935	Gain	0.93	*KIAA1217*, *PRINS*	25
**9**	28,578,490	28,747,688	169.198	Loss	0.92	*LINGO2*	173
**17**	38,756,770	39,025,634	268.864	Gain	0.93	*LOC100130581*, *ARL4D*, *DHX8*, *ETV4*	228
**2**	111,728,305	112,308,270	579.965	Gain	0.94	*LOC541471*, *ANAPC1*	365
**4**	155,866,182	155,900,189	34.007	Gain	0.91	*LRAT*	36
**21**	21,340,896	21,371,130	30.234	Loss	0.94	*NCAM2*	33
**10**	129,197,834	129,649,419	451.585	Gain	0.92	*NPS*, *FOXI2*, *CLRN3*, *PTPRE*	352
**11**	72,609,106	72,634,440	25.334	Gain	0.93	*P2RY2*	27
**4**	169,715,150	169,732,840	17.69	Gain	0.90	*PALLD*	24
**1**	162,866,812	162,891,460	24.648	Gain	0.92	*PBX1*	65
**7**	142,537,643	142,600,511	62.868	Loss	0.94	*PIP*, *TAS2R39*	45
**3**	147,574,059	147,684,016	109.957	Gain	0.92	*PLSCR2*	52
**3**	162,103,833	162,217,367	113.534	Loss	0.94	*PPM1L*	115
**17**	61,839,739	61,856,547	16.808	Loss	0.90	*PRKCA*	24
**20**	40,612,302	40,675,302	63	Loss	0.92	*PTPRT*	98
**17**	71,806,829	71,835,495	28.666	Gain	0.91	*QRICH2*, *PRPSAP1*	34
**2**	73,153,643	73,171,480	17.837	Gain	0.92	*RAB11FIP5*	24
**11**	68,019,178	68,171,177	151.999	Gain	0.90	*SAPS3*	100
**6**	76,467,807	76,531,067	63.26	Gain	0.92	*SENP6*, *MYO6*	47
**19**	43,146,190	43,168,388	22.198	Gain	0.91	*SIPA1L3*	25
**6**	45,216,097	45,240,255	24.158	Loss	0.94	*SUPT3H*	32
**22**	31,585,424	31,600,272	14.848	Gain	0.90	*SYN3*, *TIMP3*	27
**7**	139,154,242	139,203,880	49.638	Gain	0.91	*TBXAS1*	52
**2**	43,343,219	43,410,448	67.229	Loss	0.95	*THADA*	48
**7**	112,214,532	112,263,992	49.46	Gain	0.93	*TMEM168*, *C7orf60*	29
**7**	15,336,044	15,423,559	87.515	Loss	0.94	*TMEM195*	104
**12**	81,693,412	81,737,072	43.66	Loss	0.91	*TMTC2*	41
**7**	75,898,721	76,414,790	516.069	Gain	0.91	*ZP3*, *DTX2*, *FDPSL2A*, *UPK3B*, *POMZP3*	89
**1**	41,119,207	41,153,345	34.138	Gain	0.92		26
**1**	72,783,742	72,863,858	80.116	Loss	0.94		85
**1**	79,398,889	79,580,288	181.399	Loss	0.94		163
**2**	30,130,320	30,166,679	36.359	Loss	0.95		27
**2**	220,750,953	220,783,248	32.295	Gain	0.90		29
**3**	4,192,325	4,228,333	36.008	Loss	0.96		40
**3**	5,365,573	5,413,202	47.629	Loss	0.94		36
**3**	83,961,524	84,098,578	137.054	Loss	0.94		101
**3**	84,793,348	84,859,352	66.004	Gain	0.92		61
**3**	163,736,023	163,854,947	118.924	Loss	0.96		46

Table S3. *Cont.*

Chr.	Start (bp)	End (bp)	Size (Kb)	Type	Conf	Genes	Probes
**4**	63,327,869	63,365,894	38.025	Gain	0.92		25
**6**	71,955,936	72,000,380	44.444	Gain	0.90		52
**6**	141,094,351	141,937,688	843.337	Gain	0.93		595
**7**	11,276,590	11,327,466	50.876	Loss	0.95		52
**7**	20,446,450	20,484,622	38.172	Loss	0.96		34
**8**	84,326,966	84,500,231	173.265	Gain	0.93		123
**8**	90,913,791	90,962,106	48.315	Loss	0.94		37
**10**	44,523,825	44,662,300	138.475	Gain	0.91		91
**12**	82,085,657	82,139,117	53.46	Gain	0.93		48
**13**	65,259,351	65,385,772	126.421	Loss	0.94		39
**14**	42,041,583	42,155,999	114.416	Loss	0.94		90
**14**	43,890,206	43,999,036	108.83	Gain	0.93		64
**22**	20,700,074	20,901,854	201.78	Gain	0.90		152

Table S4. List of 53 CNVs identified in patients that are in-common with CNVs identified in controls. Chromosomal position (start and end), CNV size (Kb), CN type, gene(s) affected and number of probes detecting the region are noted for all CNVs.

Chr.	Start (bp)	End (bp)	Size (Kb)	Type	Conf	Genes	Probes
**2**	57,252,457	57,293,667	41.21	Gain	0.96		30
**2**	57,252,457	57,293,667	41.21	Gain	0.96		30
**2**	57,252,457	57,293,667	41.21	Gain	0.95		30
**2**	57,252,457	57,293,667	41.21	Gain	0.91		30
**3**	174,722,068	174,772,293	50.225	Gain	0.91	*NLGN1*	50
**3**	174,722,068	174,774,107	52.039	Gain	0.90	*NLGN1*	52
**3**	174,722,146	174,772,293	50.147	Gain	0.90	*NLGN1*	49
**3**	174,722,146	174,772,293	50.147	Gain	0.92	*NLGN1*	49
**4**	66,268,003	66,329,120	61.117	Gain	0.92		47
**4**	162,172,629	162,223,002	50.373	Gain	0.93		44
**5**	32,141,525	32,202,727	61.202	Gain	0.93	*PDZD2*, *GOLPH3*	56
**5**	32,141,525	32,189,791	48.266	Gain	0.92	*PDZD2*, *GOLPH3*	48
**5**	32,144,171	32,202,727	58.556	Gain	0.92	*PDZD2*, *GOLPH3*	55
**5**	32,144,171	32,202,727	58.556	Gain	0.90	*PDZD2*, *GOLPH3*	55
**5**	32,144,171	32,202,727	58.556	Gain	0.93	*PDZD2*, *GOLPH3*	55
**5**	32,144,416	32,202,727	58.311	Gain	0.93	*PDZD2*, *GOLPH3*	54
**5**	32,145,347	32,204,638	59.291	Gain	0.94	*PDZD2*, *GOLPH3*	54
**5**	32,145,347	32,198,513	53.166	Gain	0.92	*PDZD2*, *GOLPH3*	51
**5**	32,146,229	32,202,727	56.498	Gain	0.95	*PDZD2*, *GOLPH3*	52
**6**	124,468,403	124,512,504	44.101	Gain	0.91	*NKAIN2*	60
**6**	124,475,557	124,515,003	39.446	Gain	0.93	*NKAIN2*	53
**6**	124,475,557	124,514,219	38.662	Gain	0.92	*NKAIN2*	52
**6**	124,475,557	124,512,504	36.947	Gain	0.90	*NKAIN2*	51
**6**	124,475,557	124,514,219	38.662	Gain	0.92	*NKAIN2*	52
**6**	124,476,194	124,512,504	36.31	Gain	0.95	*NKAIN2*	50
**6**	124,476,194	124,512,504	36.31	Gain	0.92	*NKAIN2*	50
**8**	57,228,839	57,258,044	29.205	Gain	0.92	*PLAG1*	47

Table S4. *Cont.*

Chr.	Start (bp)	End (bp)	Size (Kb)	Type	Conf	Genes	Probes
**12**	7,883,487	8,009,418	125.931	Gain	0.91	*SLC2A14, SLC2A3*	33
**12**	7,895,002	7,985,800	90.798	Gain	0.90	*SLC2A14, SLC2A3*	28
**12**	7,895,002	8,017,944	122.942	Gain	0.91	*SLC2A14, SLC2A3*	32
**12**	7,905,243	8,015,788	110.545	Gain	0.91	*SLC2A14, SLC2A3*	27
**4**	21,656,799	21,680,565	23.766	Loss	0.91		28
**4**	21,656,799	21,680,565	23.766	Loss	0.91		28
**4**	35,051,978	35,088,582	36.604	Loss	0.95		33
**5**	99,347,328	99,473,938	126.61	Loss	0.90		66
**5**	104,461,414	104,529,373	67.959	Loss	0.92	*RAB9P1*	40
**5**	104,461,414	104,529,373	67.959	Loss	0.91	*RAB9P1*	40
**7**	8,794,054	8,831,295	37.241	Loss	0.95		35
**7**	8,794,054	8,831,295	37.241	Loss	0.96		35
**7**	8,794,054	8,831,295	37.241	Loss	0.94		35
**7**	8,794,054	8,831,295	37.241	Loss	0.94		35
**7**	8,794,054	8,831,295	37.241	Loss	0.96		35
**7**	8,794,054	8,831,295	37.241	Loss	0.92		35
**7**	141,967,332	142,005,798	38.466	Loss	0.91		40
**8**	137,751,665	137,919,630	167.965	Loss	0.92		56
**8**	137,751,665	137,919,630	167.965	Loss	0.93		56
**8**	137,751,665	137,919,630	167.965	Loss	0.90		56
**15**	85,626,838	85,671,184	44.346	Loss	0.93		48
**15**	85,626,838	85,671,184	44.346	Loss	0.92		48
**20**	14,623,339	14,780,091	156.752	Loss	0.93	*MACROD2*	238
**20**	14,727,758	14,774,064	46.306	Loss	0.95	*MACROD2*	60
**20**	14,729,522	14,774,064	44.542	Loss	0.94	*MACROD2*	57
**20**	14,816,840	14,841,243	24.403	Loss	0.95	*MACROD2*	32

Table S5. List of 386 CNVs identified in patients that are unique compared to the CNVs identified in controls. Chromosomal position (start and end), CNV size (Kb), CN type, gene(s) affected and number of probes detecting the region are noted for all CNVs.

Chr.	Start (bp)	End (bp)	Size (Kb)	Type	Conf	Genes	Probes
**1**	33,573,403	33,604,715	31.312	Gain	0.90	*PHC2*	31
**1**	40,227,064	40,271,361	44.297	Gain	0.91		34
**1**	45,936,936	46,199,969	263.033	Gain	0.91	*IPP, MAST2*	283
**1**	56,766,002	56,783,920	17.918	Gain	0.90	*PPAP2B*	29
**1**	57,582,521	57,606,826	24.305	Gain	0.91	*DAB1*	25
**1**	60,233,320	60,256,697	23.377	Gain	0.90	*C1orf87*	30
**1**	65,820,989	65,837,748	16.759	Gain	0.94	*LEPR*	33
**1**	71,759,887	71,803,596	43.709	Gain	0.91	*NEGR1*	55
**1**	82,749,313	82,786,358	37.045	Gain	0.90		29
**1**	103,901,143	104,115,323	214.18	Gain	0.90	*AMY2B, LOC648740, AMY2A, AMY1A, AMY1C, AMY1B*	27
**1**	151,020,621	151,057,372	36.751	Gain	0.91	*LCE1E, LCE1D, LCE1C, LCE1B*	27
**1**	156,716,675	156,735,109	18.434	Gain	0.91	*OR10R2*	33
**1**	158,848,020	158,874,293	26.273	Gain	0.93	*SLAMF1*	33
**1**	167,927,679	167,948,162	20.483	Gain	0.90	*SELL*	29
**1**	168,124,708	168,158,127	33.419	Gain	0.91	*SCYL3, KIFAP3*	26

Table S5. *Cont.*

Chr.	Start (bp)	End (bp)	Size (Kb)	Type	Conf	Genes	Probes
**1**	171,462,784	171,648,263	185.479	Gain	0.92		142
**1**	208,299,677	208,312,549	12.872	Gain	0.92	*SYT14*	25
**2**	18,310,984	18,381,738	70.754	Gain	0.91		35
**2**	56,456,648	56,490,949	34.301	Gain	0.90	*CCDC85A*	30
**2**	70,477,250	70,542,630	65.38	Gain	0.90	*TGFA*	29
**2**	71,794,040	71,886,259	92.219	Gain	0.91		33
**2**	73,574,651	73,618,115	43.464	Gain	0.92	*ALMS1*	35
**2**	115,458,038	115,505,177	47.139	Gain	0.90	*DPP10*	41
**2**	137,810,613	137,830,655	20.042	Gain	0.91	*THSD7B*	26
**2**	137,997,646	138,021,020	23.374	Gain	0.90	*THSD7B*	30
**2**	138,328,885	138,353,387	24.502	Gain	0.92		26
**2**	163,524,147	163,555,222	31.075	Gain	0.91		26
**2**	168,595,990	168,610,446	14.456	Gain	0.93	*STK39*	26
**2**	195,553,977	195,608,172	54.195	Gain	0.91		40
**2**	207,721,069	207,744,492	23.423	Gain	0.92	*KLF7*	25
**2**	219,798,785	219,824,021	25.236	Gain	0.96	*ATG9A, ANKZF1, GLB1L, STK16, TUBA4A*	24
**3**	56,668	657,360	600.692	Gain	0.90	*CHL1*	509
**3**	1,575,521	1,698,022	122.501	Gain	0.91		115
**3**	7,571,014	7,584,588	13.574	Gain	0.93	*GRM7*	25
**3**	19,014,033	19,041,376	27.343	Gain	0.90		31
**3**	19,016,875	19,041,376	24.501	Gain	0.91		28
**3**	19,016,875	19,041,376	24.501	Gain	0.91		28
**3**	21,228,980	21,313,310	84.33	Gain	0.90		88
**3**	23,658,694	23,817,455	158.761	Gain	0.91		52
**3**	26,904,846	26,931,601	26.755	Gain	0.91		24
**3**	35,659,921	35,720,486	60.565	Gain	0.90	*ARPP-21*	75
**3**	35,665,850	35,720,486	54.636	Gain	0.92	*ARPP-21*	68
**3**	35,692,392	35,720,486	28.094	Gain	0.90	*ARPP-21*	30
**3**	36,599,644	36,632,523	32.879	Gain	0.91		28
**3**	40,900,475	40,936,732	36.257	Gain	0.92		24
**3**	56,180,190	56,197,978	17.788	Gain	0.91	*ERC2*	25
**3**	68,319,352	68,353,779	34.427	Gain	0.90	*FAM19A1*	43
**3**	69,596,733	69,613,737	17.004	Gain	0.91		24
**3**	76,890,334	76,924,292	33.958	Gain	0.92		24
**3**	77,649,262	77,666,642	17.38	Gain	0.92	*ROBO2*	25
**3**	101,817,810	101,928,257	110.447	Gain	0.98	*GPR128, TFG*	99
**3**	101,817,810	101,928,257	110.447	Gain	0.97	*GPR128, TFG*	99
**3**	120,209,468	120,303,208	93.74	Gain	0.93	*IGSF11*	99
**3**	120,209,468	120,303,208	93.74	Gain	0.92	*IGSF11*	99
**3**	120,209,813	120,304,433	94.62	Gain	0.93	*IGSF11*	100
**3**	128,042,904	128,082,742	39.838	Gain	0.91	*CHCHD6*	33
**3**	135,621,587	135,735,170	113.583	Gain	0.91	*ANAPC13, CEP63*	29
**3**	143,303,072	143,567,869	264.797	Gain	0.91	*GK5, XRN1*	146
**3**	143,303,072	143,572,034	268.962	Gain	0.93	*GK5, XRN1*	147
**3**	143,303,072	143,567,869	264.797	Gain	0.90	*GK5, XRN1*	146
**3**	156,962,557	157,012,930	50.373	Gain	0.91	*C3orf33*	39
**3**	156,962,557	157,007,197	44.64	Gain	0.90	*C3orf33*	37

Table S5. *Cont.*

Chr.	Start (bp)	End (bp)	Size (Kb)	Type	Conf	Genes	Probes
**3**	163,361,406	163,438,398	76.992	Gain	0.93		74
**3**	164,844,550	164,873,608	29.058	Gain	0.92		24
**3**	166,762,067	166,805,923	43.856	Gain	0.91		25
**3**	171,264,089	171,471,605	207.516	Gain	0.93	*GPR160, PHC3, PRKCI*	98
**3**	171,264,089	171,466,852	202.763	Gain	0.92	*GPR160, PHC3, PRKCI*	97
**3**	174,217,958	174,291,114	73.156	Gain	0.92	*SPATA16*	82
**3**	189,058,439	189,098,718	40.279	Gain	0.93		31
**3**	189,069,317	189,088,009	18.692	Gain	0.94		26
**3**	193,216,236	193,231,084	14.848	Gain	0.91		26
**4**	26,859,619	26,881,964	22.345	Gain	0.91		30
**4**	36,054,322	36,086,761	32.439	Gain	0.91		29
**4**	42,435,788	42,476,361	40.573	Gain	0.91		30
**4**	44,664,798	44,699,744	34.946	Gain	0.92		41
**4**	44,664,798	44,699,744	34.946	Gain	0.90		41
**4**	57,744,172	57,793,124	48.952	Gain	0.90		36
**4**	60,074,423	60,248,073	173.65	Gain	0.90		105
**4**	80,402,605	80,425,296	22.691	Gain	0.91		25
**4**	81,192,534	81,216,839	24.305	Gain	0.90	*ANTXR2*	30
**4**	85,136,299	85,169,302	33.003	Gain	0.93		26
**4**	92,338,466	92,363,506	25.04	Gain	0.91	*KIAA1680*	24
**4**	92,451,901	92,486,582	34.681	Gain	0.92	*KIAA1680*	27
**4**	108,413,455	108,466,376	52.921	Gain	0.92		36
**4**	130,186,066	130,222,425	36.359	Gain	0.91	*SCLT1*	29
**4**	132,164,245	132,578,394	414.149	Gain	0.92		356
**4**	144,485,050	144,503,671	18.621	Gain	0.91	*GAB1*	24
**4**	144,936,830	145,195,551	258.721	Gain	0.92	*GYPE, GYPB*	188
**4**	145,044,238	145,081,969	37.731	Gain	0.90	*GYPE*	29
**4**	148,594,751	148,634,077	39.326	Gain	0.90	*EDNRA*	32
**4**	163,038,508	163,109,216	70.708	Gain	0.92	*FSTL5*	70
**5**	16,515,131	16,535,124	19.993	Gain	0.90	*ZNF622, FAM134B*	26
**5**	16,524,404	16,539,828	15.424	Gain	0.91	*FAM134B*	24
**5**	52,114,673	52,146,658	31.985	Gain	0.90	*PELO, ITGA1*	39
**5**	52,118,767	52,140,974	22.207	Gain	0.91	*PELO, ITGA1*	28
**5**	59,744,695	59,807,906	63.211	Gain	0.92		52
**5**	59,744,695	59,811,770	67.075	Gain	0.93		54
**5**	59,749,693	59,807,906	58.213	Gain	0.92		51
**5**	86,012,076	86,054,609	42.533	Gain	0.90		44
**5**	110,457,343	110,479,149	21.806	Gain	0.91	*WDR36*	27
**5**	111,765,498	111,798,964	33.466	Gain	0.92	*EPB41L4A, FLJ11235*	33
**5**	112,199,538	112,209,682	10.144	Gain	0.91	*APC*	29
**5**	112,201,400	112,209,829	8.429	Gain	0.91	*APC*	28
**5**	115,048,398	115,156,591	108.193	Gain	0.91		83
**5**	116,651,923	116,698,621	46.698	Gain	0.91		36
**5**	116,655,439	116,692,153	36.714	Gain	0.92		27
**5**	116,656,039	116,695,730	39.691	Gain	0.91		29
**5**	116,660,694	116,697,347	36.653	Gain	0.93		28
**5**	116,660,694	116,693,035	32.341	Gain	0.91		24
**5**	120,074,132	120,123,447	49.315	Gain	0.91		47

Table S5. *Cont.*

Chr.	Start (bp)	End (bp)	Size (Kb)	Type	Conf	Genes	Probes
**5**	128,199,361	128,294,422	95.061	Gain	0.91		98
**5**	138,968,483	139,011,408	42.925	Gain	0.90	*UBE2D2, CXXC5*	24
**5**	141,349,818	141,372,473	22.655	Gain	0.92	*GNPDA1*	24
**5**	160,496,094	160,518,978	22.884	Gain	0.92		24
**6**	18,241,646	18,345,135	103.489	Gain	0.92	*TPMT, AOF1, DEK*	83
**6**	47,928,484	47,957,746	29.262	Gain	0.91	*C6orf138*	30
**6**	49,533,193	49,561,859	28.666	Gain	0.92	*MUT, CENPQ*	29
**6**	51,919,395	51,956,146	36.751	Gain	0.91	*PKHD1*	37
**6**	63,188,441	64,153,422	964.981	Gain	0.97	*LGSN*	730
**6**	64,734,414	64,762,296	27.882	Gain	0.90	*EYS*	29
**6**	72,412,616	72,447,603	34.987	Gain	0.92		27
**6**	82,230,942	82,261,338	30.396	Gain	0.91		25
**6**	82,766,071	82,802,548	36.477	Gain	0.92		30
**6**	94,027,790	94,047,979	20.189	Gain	0.91	*EPHA7*	26
**6**	94,084,189	94,113,492	29.303	Gain	0.90	*EPHA7*	36
**6**	96,568,930	96,594,068	25.138	Gain	0.91	*FUT9*	36
**6**	124,133,684	124,171,660	37.976	Gain	0.91	*NKAIN2*	28
**6**	125,094,672	125,115,008	20.336	Gain	0.90	*NKAIN2*	30
**6**	126,795,413	126,829,371	33.958	Gain	0.91		28
**7**	8,536,565	8,583,992	47.427	Gain	0.91	*NXPH1*	50
**7**	8,549,005	8,576,446	27.441	Gain	0.93	*NXPH1*	25
**7**	8,898,081	8,939,389	41.308	Gain	0.90		37
**7**	17,153,101	17,648,404	495.303	Gain	0.90	*AHR*	446
**7**	19,580,424	19,593,018	12.594	Gain	0.90		27
**7**	26,187,682	26,213,359	25.677	Gain	0.90	*NFE2L3, HNRNPA2B1, CBX3*	26
**7**	41,151,939	41,175,803	23.864	Gain	0.92		24
**7**	75,798,663	75,822,772	24.109	Gain	0.91	*YWHAG*	29
**7**	80,092,727	80,135,656	42.929	Gain	0.90	*CD36*	55
**7**	86,460,099	86,475,960	15.861	Gain	0.91	*KIAA1324L*	27
**7**	105,671,072	105,719,044	47.972	Gain	0.92	*NAMPT*	54
**7**	105,674,698	105,716,055	41.357	Gain	0.92	*NAMPT*	47
**7**	105,677,589	105,719,044	41.455	Gain	0.90	*NAMPT*	47
**7**	111,007,466	111,052,498	45.032	Gain	0.94		25
**7**	114,211,282	114,239,605	28.323	Gain	0.91		24
**7**	145,853,816	145,886,157	32.341	Gain	0.92	*CNTNAP2*	46
**7**	145,856,756	145,886,157	29.401	Gain	0.91	*CNTNAP2*	44
**7**	147,805,976	147,831,751	25.775	Gain	0.94		26
**8**	28,781,987	28,805,704	23.717	Gain	0.91	*INTS9, HMBOX1*	27
**8**	50,594,092	50,705,323	111.231	Gain	0.94		102
**8**	87,711,543	87,739,915	28.372	Gain	0.90	*CNGB3*	36
**8**	90,838,527	90,864,652	26.125	Gain	0.90	*RIPK2*	33
**8**	95,323,546	95,339,570	16.024	Gain	0.92	*GEM*	24
**8**	117,723,882	117,890,595	166.713	Gain	0.93	*EIF3H, UTP23*	158
**8**	119,183,900	119,209,871	25.971	Gain	0.90	*EXT1*	29
**8**	120,414,388	120,438,172	23.784	Gain	0.91		27
**8**	120,419,721	120,451,773	32.052	Gain	0.91		30
**8**	120,563,544	120,611,026	47.482	Gain	0.90		31
**8**	121,863,318	121,916,778	53.46	Gain	0.90	*SNTB1*	33

Table S5. *Cont.*

Chr.	Start (bp)	End (bp)	Size (Kb)	Type	Conf	Genes	Probes
**8**	128,390,804	128,460,287	69.483	Gain	0.91		39
**8**	132,284,638	132,310,462	25.824	Gain	0.91		24
**8**	135,724,928	135,763,296	38.368	Gain	0.91	*ZFAT*	25
**8**	137,083,502	137,105,697	22.195	Gain	0.91		37
**8**	137,207,619	137,241,038	33.419	Gain	0.91		30
**8**	138,233,988	138,264,051	30.063	Gain	0.90		24
**9**	20,343,011	20,357,467	14.456	Gain	0.91	*MLLT3*	44
**9**	31,177,352	31,195,534	18.182	Gain	0.91		26
**9**	103,982,826	104,016,588	33.762	Gain	0.91		27
**9**	103,982,826	104,017,715	34.889	Gain	0.90		28
**9**	103,991,205	104,017,715	26.51	Gain	0.91		26
**10**	86,852	303,090	216.238	Gain	0.93	*ZMYND11*	190
**10**	86,852	1,680,921	1594.069	Gain	0.90	*ZMYND11, DIP2C, C10orf108, LARP4B, GTPBP4, IDI2, C10orf110, IDI1, WDR37, C10orf139, ADARB2, NCRNA00168*	837
**10**	1,158,237	1,238,647	80.41	Gain	0.91	*WDR37, C10orf139, ADARB2*	55
**10**	24,191,051	24,225,870	34.819	Gain	0.90	*KIAA1217*	46
**10**	37,456,604	37,481,448	24.844	Gain	0.90	*ANKRD30A*	24
**10**	62,204,619	62,223,459	18.84	Gain	0.90	*CDC2*	28
**10**	80,687,589	80,712,825	25.236	Gain	0.91	*ZMIZ1*	27
**10**	84,316,725	84,333,239	16.514	Gain	0.91	*NRG3*	24
**10**	84,399,731	84,421,292	21.561	Gain	0.91	*NRG3*	36
**10**	84,576,235	84,595,732	19.497	Gain	0.91	*NRG3*	27
**10**	98,968,558	99,031,965	63.407	Gain	0.90	*ARHGAP19*	42
**10**	98,974,193	99,034,317	60.124	Gain	0.90	*ARHGAP19*	40
**10**	106,003,929	106,029,361	25.432	Gain	0.90	*GSTO1, GSTO2*	27
**10**	110,672,159	110,698,179	26.02	Gain	0.91		25
**10**	119,047,876	119,074,827	26.951	Gain	0.91	*PDZD8*	31
**10**	119,776,653	119,807,671	31.018	Gain	0.90	*RAB11FIP2, CASC2*	35
**10**	123,831,207	123,854,434	23.227	Gain	0.92	*TACC2*	24
**11**	211,823	2,934,292	2722.469	Gain	0.91	*SIRT3, PSMD13, NLRP6, ATHL1, IFITM5, IFITM2, IFITM1, IFITM3, B4GALNT4, PKP3, SIGIRR, ANO9, PTDSS2, RNH1, HRAS, LRRC56, C11orf35, RASSF7, LOC143666, PHRF1, IRF7, MUPCDH, SCT, DRD4, DEAF1, TMEM80, EPS8L2, TALDO1, PDDC1, CEND1, SLC25A22, LRDD, RPLP2, SNORA52, PNPLA2, EFCAB4A, CD151, POLR2L, TSPAN4, CHID1, AP2A2, MUC6, MUC2, MUC5AC, MUC5B, TOLLIP, BRSK2, HCCA2, DUSP8, LOC338651, KRTAP5-1, KRTAP5-2, KRTAP5-3, KRTAP5-4, KRTAP5-5, FAM99A, FAM99B, KRTAP5-6, CTSD, SYT8, TNNI2, LSP1, TNNT3, MRPL23, LOC100133545, H19, IGF2, INS-IGF2, IGF2AS, INS, TH, ASCL2, C11orf21, TSPAN32, CD81, TSSC4, TRPM5, KCNQ1, KCNQ1OT1, KCNQ1DN, CDKN1C, SLC22A18AS, SLC22A18, PHLDA2, NAP1L4*	165

Table S5. *Cont.*

Chr.	Start (bp)	End (bp)	Size (Kb)	Type	Conf	Genes	Probes
**11**	15,765,333	15,791,331	25.998	Gain	0.90		30
**11**	15,770,233	15,796,302	26.069	Gain	0.92		30
**11**	15,776,946	15,795,665	18.719	Gain	0.91		24
**11**	24,503,062	24,517,108	14.046	Gain	0.91	*LUZP2*	25
**11**	26,955,953	27,194,290	238.337	Gain	0.91	*FIBIN, BBOX1*	240
**11**	27,056,207	27,096,633	40.426	Gain	0.90	*BBOX1*	46
**11**	29,547,229	29,593,722	46.493	Gain	0.90		39
**11**	29,547,756	29,593,722	45.966	Gain	0.91		37
**11**	42,847,290	42,961,216	113.926	Gain	0.91		93
**11**	57,717,749	57,747,064	29.315	Gain	0.92	*OR1S2, OR1S1*	31
**11**	59,497,196	59,538,161	40.965	Gain	0.92		24
**11**	59,785,515	59,803,006	17.491	Gain	0.91		24
**11**	78,430,159	78,451,769	21.61	Gain	0.94	*ODZ4*	31
**11**	78,686,870	78,711,812	24.942	Gain	0.91	*ODZ4*	28
**11**	81,517,502	81,564,396	46.894	Gain	0.91		30
**11**	85,335,680	85,362,876	27.196	Gain	0.93	*PICALM*	65
**11**	94,083,405	94,114,962	31.557	Gain	0.91		32
**11**	99,659,997	99,690,329	30.332	Gain	0.92	*CNTN5*	39
**11**	99,669,166	99,687,830	18.664	Gain	0.91	*CNTN5*	26
**11**	102,998,269	103,039,920	41.651	Gain	0.90		43
**11**	112,332,076	112,360,406	28.33	Gain	0.90	*NCAM1*	28
**11**	117,808,617	117,822,779	14.162	Gain	0.91	*MLL*	24
**11**	117,811,802	117,824,690	12.888	Gain	0.91	*MLL*	26
**12**	14,981,480	15,001,963	20.483	Gain	0.90	*ERP27, ARHGDIB*	25
**12**	16,469,855	16,503,960	34.105	Gain	0.91		33
**12**	16,469,855	16,503,960	34.105	Gain	0.91		33
**12**	16,476,470	16,506,851	30.381	Gain	0.92		33
**12**	90,831,618	90,859,892	28.274	Gain	0.90		36
**12**	110,657,606	110,798,825	141.219	Gain	0.93	*ACAD10, ALDH2, C12orf47, MAPKAPK5*	42
**13**	20,418,044	20,591,393	173.349	Gain	0.92	*LATS2*	103
**13**	20,418,044	20,591,393	173.349	Gain	0.93	*LATS2*	103
**13**	30,605,440	30,632,136	26.696	Gain	0.90	*HSPH1*	30
**13**	40,997,540	41,440,795	443.255	Gain	0.91	*KIAA0564*	442
**13**	45,623,679	45,645,485	21.806	Gain	0.90	*LCP1*	46
**13**	45,631,519	45,656,657	25.138	Gain	0.90	*LCP1*	51
**13**	45,638,428	45,648,003	9.575	Gain	0.91	*LCP1*	25
**13**	52,829,178	52,862,347	33.169	Gain	0.90		33
**13**	60,428,735	60,492,387	63.652	Gain	0.95		32
**13**	71,882,975	71,904,585	21.61	Gain	0.90		25
**13**	77,369,064	77,390,233	21.169	Gain	0.91	*EDNRB*	33
**13**	79,318,039	79,353,565	35.526	Gain	0.93		38
**14**	28,236,715	28,323,838	87.123	Gain	0.93	*FOXG1, C14orf23*	62
**14**	28,236,715	28,325,210	88.495	Gain	0.94	*FOXG1, C14orf23*	63
**14**	42,896,388	43,316,466	420.078	Gain	0.90		288
**14**	45,470,897	45,536,666	65.769	Gain	0.91		44
**14**	87,468,650	87,487,042	18.392	Gain	0.91	*GALC*	33
**15**	35,152,624	35,173,891	21.267	Gain	0.91	*MEIS2*	25
**15**	37,548,234	37,577,537	29.303	Gain	0.90		26
**15**	42,775,701	42,818,430	42.729	Gain	0.91	*B2M, TRIM69*	34

Table S5. *Cont.*

Chr.	Start (bp)	End (bp)	Size (Kb)	Type	Conf	Genes	Probes
**15**	42,785,893	42,818,430	32.537	Gain	0.93	*B2M, TRIM69*	29
**15**	55,193,722	55,204,013	10.291	Gain	0.91	*TCF12*	34
**15**	55,430,294	55,567,985	137.691	Gain	0.93	*CGNL1*	141
**15**	76,107,951	76,174,102	66.151	Gain	0.90	*TBC1D2B, SH2D7*	30
**15**	85,818,209	85,838,813	20.604	Gain	0.91		29
**16**	8,629,463	8,739,910	110.447	Gain	0.90	*C16orf68, ABAT*	43
**16**	22,498,570	22,689,377	190.807	Gain	0.97		49
**16**	25,330,672	25,438,375	107.703	Gain	0.92		46
**16**	25,330,672	25,438,375	107.703	Gain	0.92		46
**16**	54,792,167	54,812,699	20.532	Gain	0.90	*GNAO1*	24
**16**	63,364,955	63,389,659	24.704	Gain	0.91		33
**16**	63,369,029	63,389,029	20	Gain	0.92		30
**16**	63,369,960	63,388,189	18.229	Gain	0.90		28
**16**	63,371,038	63,397,352	26.314	Gain	0.92		38
**17**	9,909,686	9,925,857	16.171	Gain	0.91	*GAS7*	24
**17**	20,675,035	20,739,765	64.73	Gain	0.91	*CCDC144NL*	32
**17**	22,475,442	22,501,119	25.677	Gain	0.91		25
**17**	26,322,873	26,351,833	28.96	Gain	0.92	*RNF135, DPRXP4*	25
**17**	28,622,149	28,641,260	19.111	Gain	0.90	*ACCN1*	26
**17**	33,879,996	33,910,377	30.381	Gain	0.91	*ARHGAP23*	26
**17**	52,683,501	52,696,438	12.937	Gain	0.91	*MSI2*	26
**17**	52,918,407	52,926,885	8.478	Gain	0.90	*MSI2*	31
**17**	60,390,025	60,422,513	32.488	Gain	0.92	*FLJ32065*	26
**18**	50,739	945,529	894.79	Gain	0.91	*USP14, THOC1, COLEC12, CETN1, CLUL1, C18orf56, TYMS, ENOSF1, YES1, ADCYAP1*	732
**18**	4,975,533	5,216,222	240.689	Gain	0.93	*LOC642597*	189
**18**	5,000,279	5,216,222	215.943	Gain	0.92	*LOC642597*	179
**18**	5,144,387	5,299,620	155.233	Gain	0.94	*LOC642597, C18orf18, LOC339290, ZFP161*	115
**18**	5,148,405	5,183,931	35.526	Gain	0.90	*LOC642597*	26
**18**	27,901,310	27,931,250	29.94	Gain	0.98	*RNF125, RNF138*	29
**18**	27,901,310	27,931,250	29.94	Gain	0.98	*RNF125, RNF138*	29
**18**	36,448,184	36,500,713	52.529	Gain	0.91		50
**18**	56,613,840	56,649,611	35.771	Gain	0.90		33
**18**	59,110,880	59,127,173	16.293	Gain	0.91	*BCL2*	39
**19**	22,207,363	22,238,920	31.557	Gain	0.98		25
**19**	33,049,103	33,068,312	19.209	Gain	0.91		24
**19**	34,254,313	34,270,282	15.969	Gain	0.91		24
**19**	36,502,639	36,517,422	14.783	Gain	0.90	*TSHZ3*	31
**19**	39,440,516	39,460,509	19.993	Gain	0.92	*KIAA0355*	26
**19**	46,725,003	46,755,825	30.822	Gain	0.90		26
**19**	47,954,119	48,062,067	107.948	Gain	0.90	*PSG8, PSG10*	54
**19**	47,960,636	48,496,158	535.522	Gain	0.92	*PSG8, PSG10, PSG1, PSG6, PSG7, PSG11, PSG2, PSG5, PSG4, PSG9*	276
**19**	49,022,065	49,037,020	14.955	Gain	0.93	*ZNF283*	32
**19**	51,005,790	51,026,077	20.287	Gain	0.94	*RSHL1, SYMPK*	25
**19**	58,138,034	58,160,526	22.492	Gain	0.90	*ZNF816A*	29

Table S5. *Cont.*

Chr.	Start (bp)	End (bp)	Size (Kb)	Type	Conf	Genes	Probes
**20**	8,158	448,277	440.119	Gain	0.91	*DEFB125, DEFB126, DEFB127, DEFB128, DEFB129, DEFB132, C20orf96, ZCCHC3, SOX12, NRSN2, TRIB3, RBCK1, TBC1D20, CSNK2A1*	347
**20**	8,158	795,295	787.137	Gain	0.91	*DEFB125, DEFB126, DEFB127, DEFB128, DEFB129, DEFB132, C20orf96, ZCCHC3, SOX12, NRSN2, TRIB3, RBCK1, TBC1D20, CSNK2A1, TCF15, SRXN1, SCRT2, C20orf54, FAM110A*	578
**20**	10,766,941	10,789,972	23.031	Gain	0.97		30
**20**	11,935,052	11,956,711	21.659	Gain	0.90		25
**20**	15,955,845	15,977,837	21.992	Gain	0.91	*MACROD2*	30
**20**	38,740,355	38,771,128	30.773	Gain	0.91	*MAFB*	24
**20**	44,289,213	44,309,353	20.14	Gain	0.93	*CDH22*	29
**20**	44,428,961	44,442,633	13.672	Gain	0.93	*ELMO2*	24
**20**	51,618,584	51,654,796	36.212	Gain	0.91	*ZNF217*	26
**21**	36,980,079	37,015,507	35.428	Gain	0.90	*SIM2*	29
**22**	25,203,174	25,219,492	16.318	Gain	0.90	*HPS4, SRRD, TFIP11*	26
**22**	27,978,142	28,002,545	24.403	Gain	0.92	*EMID1, RHBDD3, EWSR1*	25
**22**	33,096,582	33,112,138	15.556	Gain	0.91		39
**22**	33,694,678	33,720,051	25.373	Gain	0.90		35
**1**	34,667,546	34,813,033	145.487	Loss	0.92		92
**1**	82,801,000	82,821,932	20.932	Loss	0.93		31
**1**	82,801,000	82,821,932	20.932	Loss	0.94		31
**1**	187,592,652	187,810,312	217.66	Loss	0.92		157
**1**	189,990,762	190,023,005	32.243	Loss	0.91		34
**2**	22,087,558	22,261,901	174.343	Loss	0.91		110
**2**	22,087,558	22,261,901	174.343	Loss	0.93		110
**2**	35,671,632	35,943,433	271.801	Loss	0.92		215
**2**	95,821,582	96,060,997	239.415	Loss	0.92	*LOC729234, GPAT2*	60
**2**	133,536,392	133,554,866	18.474	Loss	0.90	*NAP5*	26
**2**	215,167,158	215,204,595	37.437	Loss	0.93		49
**2**	215,167,158	215,204,595	37.437	Loss	0.91		49
**3**	2,492,115	2,561,010	68.895	Loss	0.93	*CNTN4*	101
**3**	2,492,115	2,561,010	68.895	Loss	0.93	*CNTN4*	101
**3**	6,562,398	6,603,706	41.308	Loss	0.94		42
**3**	6,562,398	6,603,706	41.308	Loss	0.93		42
**3**	21,273,619	21,339,035	65.416	Loss	0.92		62
**3**	22,537,178	22,583,488	46.31	Loss	0.93		46
**3**	82,794,687	82,851,969	57.282	Loss	0.92		47
**3**	166,523,809	166,565,186	41.377	Loss	0.95		39
**3**	166,525,250	166,565,186	39.936	Loss	0.93		38
**3**	177,370,126	177,396,832	26.706	Loss	0.93		26
**3**	177,370,126	177,396,832	26.706	Loss	0.94		26
**3**	177,370,126	177,396,832	26.706	Loss	0.96		26
**3**	177,370,126	177,399,625	29.499	Loss	0.93		27
**3**	177,370,126	177,396,832	26.706	Loss	0.93		26

Table S5. *Cont.*

Chr.	Start (bp)	End (bp)	Size (Kb)	Type	Conf	Genes	Probes
**4**	36,592,048	36,613,184	21.136	Loss	0.94		24
**4**	71,210,205	71,228,042	17.837	Loss	0.90		32
**4**	179,774,311	179,815,206	40.895	Loss	0.91		33
**5**	12,729,293	12,791,916	62.623	Loss	0.93		57
**5**	17,653,401	17,794,277	140.876	Loss	0.92		86
**5**	21,226,481	21,475,598	249.117	Loss	0.96		124
**5**	59,226,765	59,304,137	77.372	Loss	0.94		53
**5**	61,460,851	61,504,678	43.827	Loss	0.95		31
**5**	61,460,851	61,504,678	43.827	Loss	0.93		31
**5**	101,501,564	101,562,374	60.81	Loss	0.93		25
**5**	111,367,616	111,425,094	57.478	Loss	0.93		46
**5**	120,452,412	120,495,551	43.139	Loss	0.90		28
**5**	120,632,046	120,679,136	47.09	Loss	0.94		46
**6**	5,606,114	5,656,830	50.716	Loss	0.95	*FARS2*	25
**7**	16,315,652	16,368,475	52.823	Loss	0.91	*LOC729920*	82
**7**	17,079,505	17,145,713	66.208	Loss	0.94		67
**7**	40,405,914	40,448,594	42.68	Loss	0.91	*C7orf10*	54
**7**	42,329,458	42,358,467	29.009	Loss	0.92		30
**7**	84,986,163	85,035,654	49.491	Loss	0.94		41
**7**	92,319,307	92,343,906	24.599	Loss	0.94		26
**7**	92,319,307	92,343,906	24.599	Loss	0.94		26
**7**	110,748,452	111,047,157	298.705	Loss	0.93	*IMMP2L*	291
**7**	110,752,225	110,872,864	120.639	Loss	0.90	*IMMP2L*	140
**7**	112,804,639	112,843,105	38.466	Loss	0.91		28
**7**	125,393,915	125,565,955	172.04	Loss	0.92		160
**8**	16,500,333	16,571,629	71.296	Loss	0.90		80
**8**	75,713,501	75,766,716	53.215	Loss	0.96		50
**8**	82,493,081	82,617,455	124.374	Loss	0.93	*PMP2*, *FABP9*, *FABP4*, *FABP12*	61
**9**	25,249,407	25,339,934	90.527	Loss	0.93		52
**9**	28,451,776	28,560,165	108.389	Loss	0.95	*LINGO2*	70
**9**	29,406,148	29,492,782	86.634	Loss	0.94		44
**9**	30,420,351	30,550,202	129.851	Loss	0.95		72
**9**	76,397,394	76,433,949	36.555	Loss	0.94	*RORB*	25
**9**	104,331,902	104,396,632	64.73	Loss	0.96		35
**9**	104,331,902	104,396,632	64.73	Loss	0.96		35
**10**	27,646,608	27,745,736	99.128	Loss	0.92	*PTCHD3*	77
**10**	53,053,500	53,076,874	23.374	Loss	0.92	*PRKG1*	33
**10**	67,744,533	67,785,552	41.019	Loss	0.95	*CTNNA3*	48
**10**	67,744,533	67,785,552	41.019	Loss	0.91	*CTNNA3*	48
**10**	67,771,483	67,830,676	59.193	Loss	0.94	*CTNNA3*	80
**10**	68,018,982	68,043,924	24.942	Loss	0.91	*CTNNA3*	35
**10**	83,632,244	83,956,625	324.381	Loss	0.93	*NRG3*	535
**10**	92,367,327	92,470,718	103.391	Loss	0.91		125
**10**	107,975,297	108,005,972	30.675	Loss	0.92		41
**11**	4,755,621	4,866,656	111.035	Loss	0.95	*OR52R1*, *OR51F2*, *OR51S1*, *OR51T1*	69
**11**	24,089,992	24,181,255	91.263	Loss	0.91		63
**11**	31,278,439	31,334,153	55.714	Loss	0.94	*DCDC1*	35
**11**	31,278,439	31,334,153	55.714	Loss	0.96	*DCDC1*	35

Table S5. *Cont.*

Chr.	Start (bp)	End (bp)	Size (Kb)	Type	Conf	Genes	Probes
**11**	37,984,236	38,072,388	88.152	Loss	0.92		46
**11**	95,844,428	95,917,476	73.048	Loss	0.91		54
**12**	58,222,020	58,358,584	136.564	Loss	0.94		73
**13**	63,303,320	63,832,472	529.152	Loss	0.92		244
**13**	69,670,394	69,796,605	126.211	Loss	0.95		38
**13**	91,112,192	91,194,758	82.566	Loss	0.96	*GPC5*	88
**13**	95,191,736	95,231,868	40.132	Loss	0.93	*DNAJC3*	49
**14**	34,643,612	34,745,141	101.529	Loss	0.92	*PPP2R3C*, *KIAA0391*	57
**14**	34,643,612	34,752,981	109.369	Loss	0.92	*PPP2R3C*, *KIAA0391*	59
**14**	44,251,826	44,294,996	43.17	Loss	0.93		45
**14**	47,299,871	47,347,026	47.155	Loss	0.93		35
**15**	32,717,275	32,769,870	52.595	Loss	0.92		63
**16**	21,504,017	21,680,369	176.352	Loss	0.92	*METTL9*, *IGSF6*, *OTOA*	98
**17**	19,439,549	19,478,897	39.348	Loss	0.90		29
**17**	48,308,591	48,671,069	362.478	Loss	0.91		444
**18**	1,896,471	1,974,869	78.398	Loss	0.92		62
**18**	63,997,797	64,044,740	46.943	Loss	0.93		51
**19**	37,026,345	37,055,315	28.97	Loss	0.91		39

Table S6. List of 317 genes associated with CNVs uniquely identified in patients (compared to controls) analyzed in WebGestalt software.

Gene Symbol	Gene Name
ABAT	4-aminobutyrate aminotransferase
ACAD10	acyl-CoA dehydrogenase family, member 10
ADARB2	adenosine deaminase, RNA-specific, B2
ADCYAP1	adenylate cyclase activating polypeptide 1 (pituitary)
AHR	aryl hydrocarbon receptor
ALDH2	aldehyde dehydrogenase 2 family (mitochondrial)
ALMS1	Alstrom syndrome 1
AMY1A	amylase, alpha 1A (salivary)
AMY1B	amylase, alpha 1B (salivary)
AMY1C	amylase, alpha 1C (salivary)
AMY2A	amylase, alpha 2A (pancreatic)
AMY2B	amylase, alpha 2B (pancreatic)
ANAPC13	anaphase promoting complex subunit 13
ANKRD30A	ankyrin repeat domain 30A
ANKZF1	ankyrin repeat and zinc finger domain containing 1
ANO9	anoctamin 9
ANTXR2	anthrax toxin receptor 2
AP2A2	adaptor-related protein complex 2, alpha 2 subunit
APC	adenomatous polyposis coli
ARHGAP19	Rho GTPase activating protein 19
ARHGAP23	Rho GTPase activating protein 23
ARHGDIB	Rho GDP dissociation inhibitor (GDI) beta

Table S6. *Cont.*

Gene Symbol	Gene Name
ASCL2	achaete-scute complex homolog 2 (Drosophila)
ATG9A	autophagy related 9A
ATHL1	ATH1, acid trehalase-like 1 (yeast)
B2M	beta-2-microglobulin
B4GALNT4	beta-1,4-*N*-acetyl-galactosaminyl transferase 4
BBOX1	butyrobetaine (gamma), 2-oxoglutarate dioxygenase (gamma-butyrobetaine hydroxylase) 1
BCL2	B-cell CLL/lymphoma 2
BRSK2	BR serine/threonine kinase 2
C10orf108	chromosome 10 open reading frame 108
C11orf21	chromosome 11 open reading frame 21
C11orf35	chromosome 11 open reading frame 35
C14orf23	chromosome 14 open reading frame 23
C18orf56	chromosome 18 open reading frame 56
C1orf87	chromosome 1 open reading frame 87
C20orf96	chromosome 20 open reading frame 96
C3orf33	chromosome 3 open reading frame 33
CASC2	cancer susceptibility candidate 2 (non-protein coding)
CBX3	chromobox homolog 3
CCDC144NL	coiled-coil domain containing 144 family, N-terminal like
CCDC85A	coiled-coil domain containing 85A
CD151	CD151 molecule (Raph blood group)
CD36	CD36 molecule (thrombospondin receptor)
CD81	CD81 molecule
CDH22	cadherin 22, type 2
CDKN1C	cyclin-dependent kinase inhibitor 1C (p57, Kip2)
CEND1	cell cycle exit and neuronal differentiation 1
CENPQ	centromere protein Q
CEP63	centrosomal protein 63 kDa
CETN1	centrin, EF-hand protein, 1
CGNL1	cingulin-like 1
CHCHD6	coiled-coil-helix-coiled-coil-helix domain containing 6
CHID1	chitinase domain containing 1
CHL1	cell adhesion molecule with homology to L1CAM (close homolog of L1)
CLUL1	clusterin-like 1 (retinal)
CNGB3	cyclic nucleotide gated channel beta 3
CNTN4	contactin 4
CNTN5	contactin 5
CNTNAP2	contactin associated protein-like 2
COLEC12	collectin sub-family member 12
CSNK2A1	casein kinase 2, alpha 1 polypeptide
CTNNA3	catenin (cadherin-associated protein), alpha 3
CTSD	cathepsin D
CXXC5	CXXC finger protein 5
DAB1	disabled homolog 1 (Drosophila)

Table S6. *Cont.*

Gene Symbol	Gene Name
DCDC1	doublecortin domain containing 1
DEAF1	deformed epidermal autoregulatory factor 1 (Drosophila)
DEFB125	defensin, beta 125
DEFB126	defensin, beta 126
DEFB127	defensin, beta 127
DEFB128	defensin, beta 128
DEFB129	defensin, beta 129
DEFB132	defensin, beta 132
DEK	DEK oncogene
DIP2C	DIP2 disco-interacting protein 2 homolog C (Drosophila)
DNAJC3	DnaJ (Hsp40) homolog, subfamily C, member 3
DPP10	dipeptidyl-peptidase 10 (non-functional)
DPRXP4	divergent-paired related homeobox pseudogene 4
DRD4	dopamine receptor D4
DUSP8	dual specificity phosphatase 8
EDNRA	endothelin receptor type A
EDNRB	endothelin receptor type B
EFCAB4A	EF-hand calcium binding domain 4A
EIF3H	eukaryotic translation initiation factor 3, subunit H
ELMO2	engulfment and cell motility 2
EMID1	EMI domain containing 1
ENOSF1	enolase superfamily member 1
EPB41L4A	erythrocyte membrane protein band 4.1 like 4A
EPHA7	EPH receptor A7
EPS8L2	EPS8-like 2
ERC2	ELKS/RAB6-interacting/CAST family member 2
ERP27	endoplasmic reticulum protein 27
EWSR1	Ewing sarcoma breakpoint region 1
EXT1	exostosin 1
EYS	eyes shut homolog (Drosophila)
FABP12	fatty acid binding protein 12
FABP4	fatty acid binding protein 4, adipocyte
FABP9	fatty acid binding protein 9, testis
FAM110A	family with sequence similarity 110, member A
FAM134B	family with sequence similarity 134, member B
FAM19A1	family with sequence similarity 19 (chemokine (C-C motif)-like), member A1
FAM99A	family with sequence similarity 99, member A (non-protein coding)
FAM99B	family with sequence similarity 99, member B (non-protein coding)
FARS2	phenylalanyl-tRNA synthetase 2, mitochondrial
FIBIN	fin bud initiation factor homolog (zebrafish)
FLJ11235	uncharacterized FLJ11235
FOXG1	forkhead box G1
FSTL5	follistatin-like 5
FUT9	fucosyltransferase 9 (alpha (1,3) fucosyltransferase)

Table S6. *Cont.*

Gene Symbol	Gene Name
GAB1	GRB2-associated binding protein 1
GALC	galactosylceramidase
GAS7	growth arrest-specific 7
GEM	GTP binding protein overexpressed in skeletal muscle
GK5	glycerol kinase 5 (putative)
GLB1L	galactosidase, beta 1-like
GNAO1	guanine nucleotide binding protein (G protein), alpha activating activity polypeptide O
GNPDA1	glucosamine-6-phosphate deaminase 1
GPAT2	glycerol-3-phosphate acyltransferase 2, mitochondrial
GPC5	glypican 5
GPR128	G protein-coupled receptor 128
GPR160	G protein-coupled receptor 160
GRM7	glutamate receptor, metabotropic 7
GSTO1	glutathione S-transferase omega 1
GSTO2	glutathione S-transferase omega 2
GTPBP4	GTP binding protein 4
GYPB	glycophorin B (MNS blood group)
GYPE	glycophorin E (MNS blood group)
H19	H19, imprinted maternally expressed transcript (non-protein coding)
HMBOX1	homeobox containing 1
HNRNPA2B1	heterogeneous nuclear ribonucleoprotein A2/B1
HPS4	Hermansky-Pudlak syndrome 4
HRAS	v-Ha-ras Harvey rat sarcoma viral oncogene homolog
HSPH1	heat shock 105 kDa/110 kDa protein 1
IDI1	isopentenyl-diphosphate delta isomerase 1
IDI2	isopentenyl-diphosphate delta isomerase 2
IFITM1	interferon induced transmembrane protein 1
IFITM2	interferon induced transmembrane protein 2
IFITM3	interferon induced transmembrane protein 3
IFITM5	interferon induced transmembrane protein 5
IGF2	insulin-like growth factor 2 (somatomedin A)
IGSF11	immunoglobulin superfamily, member 11
IGSF6	immunoglobulin superfamily, member 6
IMMP2L	IMP2 inner mitochondrial membrane peptidase-like (*S. cerevisiae*)
INS	insulin
INS-IGF2	INS-IGF2 readthrough
INTS9	integrator complex subunit 9
IPP	intracisternal A particle-promoted polypeptide
IRF7	interferon regulatory factor 7
ITGA1	integrin, alpha 1
KCNQ1	potassium voltage-gated channel, KQT-like subfamily, member 1
KCNQ1DN	KCNQ1 downstream neighbor (non-protein coding)
KCNQ1OT1	KCNQ1 opposite strand/antisense transcript 1 (non-protein coding)
KIAA0355	KIAA0355

Table S6. *Cont.*

Gene Symbol	Gene Name
KIAA0391	KIAA0391
KIAA1324L	KIAA1324-like
KIFAP3	kinesin-associated protein 3
KLF7	Kruppel-like factor 7 (ubiquitous)
KRTAP5-1	keratin associated protein 5-1
KRTAP5-2	keratin associated protein 5-2
KRTAP5-3	keratin associated protein 5-3
KRTAP5-4	keratin associated protein 5-4
KRTAP5-5	keratin associated protein 5-5
KRTAP5-6	keratin associated protein 5-6
LARP4B	La ribonucleoprotein domain family, member 4B
LATS2	LATS, large tumor suppressor, homolog 2 (Drosophila)
LCE1B	late cornified envelope 1B
LCE1C	late cornified envelope 1C
LCE1D	late cornified envelope 1D
LCE1E	late cornified envelope 1E
LCP1	lymphocyte cytosolic protein 1 (L-plastin)
LEPR	leptin receptor
LGSN	lengsin, lens protein with glutamine synthetase domain
LOC143666	uncharacterized LOC143666
LOC338651	uncharacterized LOC338651
LRRC56	leucine rich repeat containing 56
LSP1	lymphocyte-specific protein 1
LUZP2	leucine zipper protein 2
MAFB	v-maf musculoaponeurotic fibrosarcoma oncogene homolog B (avian)
MAPKAPK5	mitogen-activated protein kinase-activated protein kinase 5
MAST2	microtubule associated serine/threonine kinase 2
MEIS2	Meis homeobox 2
METTL9	methyltransferase like 9
MLL	myeloid/lymphoid or mixed-lineage leukemia (trithorax homolog, Drosophila)
MLLT3	myeloid/lymphoid or mixed-lineage leukemia (trithorax homolog, Drosophila); translocated to, 3
MRPL23	mitochondrial ribosomal protein L23
MSI2	musashi homolog 2 (Drosophila)
MUC2	mucin 2, oligomeric mucus/gel-forming
MUC5AC	mucin 5AC, oligomeric mucus/gel-forming
MUC5B	mucin 5B, oligomeric mucus/gel-forming
MUC6	mucin 6, oligomeric mucus/gel-forming
MUT	methylmalonyl CoA mutase
NAMPT	nicotinamide phosphoribosyltransferase
NAP1L4	nucleosome assembly protein 1-like 4
NCAM1	neural cell adhesion molecule 1
NEGR1	neuronal growth regulator 1
NFE2L3	nuclear factor (erythroid-derived 2)-like 3
NLRP6	NLR family, pyrin domain containing 6

Table S6. *Cont.*

Gene Symbol	Gene Name
NRG3	neuregulin 3
NRSN2	neurensin 2
NXPH1	neurexophilin 1
ODZ4	odz, odd Oz/ten-m homolog 4 (Drosophila)
OR10R2	olfactory receptor, family 10, subfamily R, member 2
OR1S1	olfactory receptor, family 1, subfamily S, member 1
OR1S2	olfactory receptor, family 1, subfamily S, member 2
OR51F2	olfactory receptor, family 51, subfamily F, member 2
OR51S1	olfactory receptor, family 51, subfamily S, member 1
OR51T1	olfactory receptor, family 51, subfamily T, member 1
OR52R1	olfactory receptor, family 52, subfamily R, member 1
OTOA	otoancorin
PDDC1	Parkinson disease 7 domain containing 1
PDZD8	PDZ domain containing 8
PELO	pelota homolog (Drosophila)
PHC2	polyhomeotic homolog 2 (Drosophila)
PHC3	polyhomeotic homolog 3 (Drosophila)
PHLDA2	pleckstrin homology-like domain, family A, member 2
PHRF1	PHD and ring finger domains 1
PICALM	phosphatidylinositol binding clathrin assembly protein
PKHD1	polycystic kidney and hepatic disease 1 (autosomal recessive)
PKP3	plakophilin 3
PMP2	peripheral myelin protein 2
PNPLA2	patatin-like phospholipase domain containing 2
POLR2L	polymerase (RNA) II (DNA directed) polypeptide L, 7.6 kDa
PPAP2B	phosphatidic acid phosphatase type 2B
PPP2R3C	protein phosphatase 2, regulatory subunit B", gamma
PRKCI	protein kinase C, iota
PRKG1	protein kinase, cGMP-dependent, type I
PSG1	pregnancy specific beta-1-glycoprotein 1
PSG11	pregnancy specific beta-1-glycoprotein 11
PSG2	pregnancy specific beta-1-glycoprotein 2
PSG4	pregnancy specific beta-1-glycoprotein 4
PSG5	pregnancy specific beta-1-glycoprotein 5
PSG6	pregnancy specific beta-1-glycoprotein 6
PSG7	pregnancy specific beta-1-glycoprotein 7 (gene/pseudogene)
PSG8	pregnancy specific beta-1-glycoprotein 8
PSG9	pregnancy specific beta-1-glycoprotein 9
PSMD13	proteasome (prosome, macropain) 26S subunit, non-ATPase, 13
PTCHD3	patched domain containing 3
PTDSS2	phosphatidylserine synthase 2
RAB11FIP2	RAB11 family interacting protein 2 (class I)
RASSF7	Ras association (RalGDS/AF-6) domain family (*N*-terminal) member 7
RBCK1	RanBP-type and C3HC4-type zinc finger containing 1

Table S6. *Cont.*

Gene Symbol	Gene Name
RHBDD3	rhomboid domain containing 3
RIPK2	receptor-interacting serine-threonine kinase 2
RNF125	ring finger protein 125, E3 ubiquitin protein ligase
RNF135	ring finger protein 135
RNF138	ring finger protein 138, E3 ubiquitin protein ligase
RNH1	ribonuclease/angiogenin inhibitor 1
ROBO2	roundabout, axon guidance receptor, homolog 2 (Drosophila)
RORB	RAR-related orphan receptor B
RPLP2	ribosomal protein, large, P2
SCLT1	sodium channel and clathrin linker 1
SCRT2	scratch homolog 2, zinc finger protein (Drosophila)
SCT	secretin
SCYL3	SCY1-like 3 (*S. cerevisiae*)
SELL	selectin L
SH2D7	SH2 domain containing 7
SIGIRR	single immunoglobulin and toll-interleukin 1 receptor (TIR) domain
SIM2	single-minded homolog 2 (Drosophila)
SIRT3	sirtuin 3
SLAMF1	signaling lymphocytic activation molecule family member 1
SLC22A18	solute carrier family 22, member 18
SLC22A18AS	solute carrier family 22 (organic cation transporter), member 18 antisense
SLC25A22	solute carrier family 25 (mitochondrial carrier: glutamate), member 22
SNORA52	small nucleolar RNA, H/ACA box 52
SNTB1	syntrophin, beta 1 (dystrophin-associated protein A1, 59 kDa, basic component 1)
SOX12	SRY (sex determining region Y)-box 12
SPATA16	spermatogenesis associated 16
SRRD	SRR1 domain containing
SRXN1	sulfiredoxin 1
STK16	serine/threonine kinase 16
STK39	serine threonine kinase 39
SYMPK	symplekin
SYT14	synaptotagmin XIV
SYT8	synaptotagmin VIII
TACC2	transforming, acidic coiled-coil containing protein 2
TALDO1	transaldolase 1
TBC1D20	TBC1 domain family, member 20
TBC1D2B	TBC1 domain family, member 2B
TCF12	transcription factor 12
TCF15	transcription factor 15 (basic helix-loop-helix)
TFG	TRK-fused gene
TFIP11	tuftelin interacting protein 11
TGFA	transforming growth factor, alpha
TH	tyrosine hydroxylase
THOC1	THO complex 1

Table S6. *Cont.*

Gene Symbol	Gene Name
THSD7B	thrombospondin, type I, domain containing 7B
TMEM80	transmembrane protein 80
TNNI2	troponin I type 2 (skeletal, fast)
TNNT3	troponin T type 3 (skeletal, fast)
TOLLIP	toll interacting protein
TPMT	thiopurine S-methyltransferase
TRIB3	tribbles homolog 3 (Drosophila)
TRIM69	tripartite motif containing 69
TRPM5	transient receptor potential cation channel, subfamily M, member 5
TSHZ3	teashirt zinc finger homeobox 3
TSPAN32	tetraspanin 32
TSPAN4	tetraspanin 4
TSSC4	tumor suppressing subtransferable candidate 4
TUBA4A	tubulin, alpha 4a
TYMS	thymidylate synthetase
UBE2D2	ubiquitin-conjugating enzyme E2D 2
USP14	ubiquitin specific peptidase 14 (tRNA-guanine transglycosylase)
UTP23	UTP23, small subunit (SSU) processome component, homolog (yeast)
WDR36	WD repeat domain 36
WDR37	WD repeat domain 37
XRN1	5'–3' exoribonuclease 1
YES1	v-yes-1 Yamaguchi sarcoma viral oncogene homolog 1
YWHAG	tyrosine 3-monooxygenase/tryptophan 5-monooxygenase activation protein, gamma polypeptide
ZCCHC3	zinc finger, CCHC domain containing 3
ZFAT	zinc finger and AT hook domain containing
ZFP161	zinc finger protein 161 homolog (mouse)
ZMIZ1	zinc finger, MIZ-type containing 1
ZMYND11	zinc finger, MYND-type containing 11
ZNF217	zinc finger protein 217
ZNF283	zinc finger protein 283
ZNF622	zinc finger protein 622

Table S7. List of 114 miRs with sequences complementary to the enriched 3' UTR target region of genes associated with CNVs uniquely identified in patients (compared to controls).

miRs				
hsa-miR-101	hsa-miR-103	hsa-miR-186	hsa-miR-373	hsa-miR-526B
hsa-miR-106A	hsa-miR-106B	hsa-miR-18B	hsa-miR-374	hsa-miR-93
hsa-miR-107	hsa-miR-122A	hsa-miR-195	hsa-miR-377	
hsa-miR-126	hsa-miR-124A	hsa-miR-196A	hsa-miR-381	
hsa-miR-133B	hsa-miR-133A	hsa-miR-196B	hsa-miR-424	
hsa-miR-135A	hsa-miR-137	hsa-miR-199A	hsa-miR-448	
hsa-miR-135B	hsa-miR-139	hsa-miR-202	hsa-miR-452	

Table S7. *Cont.*

**miRs**			
hsa-miR-141	**hsa-miR-142-5P**	**hsa-miR-204**	**hsa-miR-485-3P**
hsa-miR-142-3P	hsa-miR-148A	hsa-miR-20B	hsa-miR-485-5P
hsa-miR-155	hsa-miR-148B	hsa-miR-211	hsa-miR-489
hsa-miR-15A	hsa-miR-152	hsa-miR-219	hsa-miR-496
hsa-miR-15B	hsa-miR-154	hsa-miR-23B	hsa-miR-497
hsa-miR-17-5P	hsa-miR-16	hsa-miR-25	hsa-miR-506
hsa-miR-181B	hsa-miR-181A	hsa-miR-29A	hsa-miR-514
hsa-miR-181C	hsa-miR-181D	hsa-miR-29B	hsa-miR-517B
hsa-miR-183	hsa-miR-182	hsa-miR-29C	hsa-miR-518A-2
hsa-miR-18A	hsa-miR-218	hsa-miR-302C	hsa-miR-519A
hsa-miR-200A	hsa-miR-302B	hsa-miR-302D	hsa-miR-519B
hsa-miR-200B	hsa-miR-30A-5P	hsa-miR-30A-3P	hsa-miR-519C
hsa-miR-200C	hsa-miR-31	hsa-miR-30D	hsa-miR-519D
hsa-miR-203	hsa-miR-32	hsa-miR-30E-3P	hsa-miR-520A
hsa-miR-20A	hsa-miR-34B	hsa-miR-30E-5P	hsa-miR-520B
hsa-miR-221	hsa-miR-429	hsa-miR-326	hsa-miR-520C
hsa-miR-222	hsa-miR-432	hsa-miR-330	hsa-miR-520D
hsa-miR-23A	hsa-miR-493	hsa-miR-363	hsa-miR-520E
hsa-miR-302A	hsa-miR-9	hsa-miR-367	hsa-miR-520F
hsa-miR-30B	hsa-miR-92	hsa-miR-369-3P	hsa-miR-522
hsa-miR-30C	hsa-miR-96	hsa-miR-372	hsa-miR-524
